# mRNA caps accumulate in stress granules and are essential for their formation

**DOI:** 10.1007/s00018-025-05947-8

**Published:** 2025-11-25

**Authors:** Mingxin Guo, Ying Feng, Guantong Qi, Wenran Ma, Xiaoli He, Ruiqi Li, Yu Liu, Jin Wang

**Affiliations:** State Key Laboratory of Reproductive Regulation and Breeding of Grassland Livestock, Institute of Biomedical Sciences, School of Life Sciences, Inner Mongolia University, Hohhot, 010030 People’s Republic of China

**Keywords:** Stress granule, mRNA cap, CapQuant, Stress

## Abstract

Stress granules (SGs) are essential cytoplasmic, membraneless organelles that form in response to cellular stress, functioning to prevent mRNA translation and protect mRNA from damage. However, the mechanism of SG formation remains largely unknown. Here, utilizing a systems-level technique for quantification of RNA cap epitranscriptome, we found that mRNA Cap1 and non-canonical caps are predominantly enriched in SGs, with the composition of mRNA caps in SGs of mammalian cells differing between different stress conditions. Knockdown of RNA guanine-7-methyltransferase (RNMT) and phosphorylated CTD interacting factor 1(PCIF1) both resulted in substantial changes in the content and composition of mRNA caps and RNMT knockdown caused failure of SG formation under different stress conditions. Furthermore, proteomic, Co-IP and confocal immunofluorescence analysis of these knockdown cells and SGs revealed that mRNAs partition into SGs through cap-protein interactions. These findings collectively revealed the significant role of mRNA cap in SG formation and stress response in mammalian cells.

**Supplementary Information:**

The online version contains supplementary material available at 10.1007/s00018-025-05947-8.

## Introduction

Stress granules (SGs) are crucial cytoplasmic, membraneless organelles [1, 2] that arise in response to cellular stress [3, 4]. They play a pivotal role in modulating mRNA translation and degradation under a variety of stress conditions, such as heat shock, oxidative stress, and UV, thereby helping cells to adapt and survive under adverse conditions [5]. SG formation is a multifaceted process influenced by a variety of factors. Specifically, it is believed to involve the assembly of untranslated mRNA molecules, ribosomal subunits, and a spectrum of proteins. These proteins include RNA-binding proteins that can directly interact with mRNA molecules, as well as translation initiation factors that play a critical role in the early stages of protein synthesis. Recent findings revealed mRNAs’ significant role in cellular stress responses, and that characteristics of mRNA such as length, structural complexity, and translation efficiency influence its enrichment in SGs [6]. The GTPase activating protein (SH3 domain) binding protein 1 (G3BP1) protein functions as an essential molecular switch governing SG formation during cellular stress [7, 8], and has been established as a canonical marker protein in SG studies.

mRNA is the core component of SG RNA, comprising over 85% of SG total RNA. Studies have showed that nearly all mRNAs could potentially be targeted to SGs, with targeting efficiencies varying widely, from less than 1% to over 95%[6, 9]. One study utilizing RNA sequencing (RNA-seq) analysis of purified SG cores and single-molecule fluorescence in situ hybridization (smFISH) verification found that 10% of total mRNA molecules accumulate in mammalian SGs, with 185 genes having over 50% of their mRNA molecules localized within SGs [6].

The 5’ cap is a crucial RNA modification that plays a significant role in RNA stability, metabolism, and function [10]. The classic cap structure consists of an *N*7-methylguanosine (m^7^G) linked to the 5’ end of mRNA adjacent to the first transcribed nucleotide [11]. In higher eukaryotes, the 5’ penultimate nucleotides in different m^7^GpppNs (cap 0) can be 2’-*O*-methylated to generate different m^7^GpppNm (cap 1) structures [12]. Notably, recent studies have identified non-canonical ‘metabolite caps’, which refer to cellular metabolites such as NAD and FAD, covalently attached to RNA 5’ends, potentially linking cellular metabolic states to RNA regulation [13, 14]. These diverse capping mechanisms highlight the complexity of RNA 5’-end modifications in biological processes. The 5’ cap protects mRNA from degradation and controls its cellular fate through specific interactions with various proteins [15, 16], thereby influencing translation efficiency and mRNA decay. The 5’ cap could potentially influence mRNA recruitment to SGs, thereby affecting the cellular stress response [17]. Despite the significance of RNA modifications like m^6^A [18] and m^6^Am [19] in SGs, studies on the global landscape of RNA modifications, particularly the 5′ caps within SGs, remain limited. Through understanding how mRNA caps change when cells are stressed and whether and how they contribute to SG formation, we can gain a more comprehensive understanding of how cells regulate their gene expression under stress. This knowledge is essential for uncovering the molecular underpinnings of stress responses and may reveal new avenues for modulating these processes in various pathological conditions.

In the current study, by using CapQuant [20], an innovative system-level mass spectrometry method we recently developed that enables accurate and sensitive quantification of the RNA cap epitranscriptome of any organism, we mapped the mRNA cap landscape of human osteosarcoma cells and SGs formed in these cells under several stresses. The resulting data revealed substantial variations in both the composition and content of mRNA caps within SGs, which is heavily stress type-dependent. Additionally, knockdown of the cap methyltransferases RNMT and PCIF1 both led to notable changes in cap composition and RNMT knockdown failure of SG formation under diverse stress conditions. Furthermore, proteomic analysis of these knockdown cells and SGs suggested that mRNA Cap1 is involved in SG formation, at least partially, through interacting with certain proteins that partition into SGs along with the mRNA.

## Materials and methods

### Cell culture

Human osteosarcoma U-2 OS (FuHeng BioLogy) was cultured with DMEM high glucose medium (containing 10% fetal bovine serum and 1% penicillin/streptomycin) at 37 ℃ and 5% CO_2_.

### Stressing cells

The cell coverage reached 85% or more, the medium was replaced with a new medium and incubated for 1 h. This was followed by stress treatments: 0.5 mM NaAsO_2_ treatment for 1 h; heat shock (HS) at 45 °C for 1 h; or UVA (365 nm) ultraviolet irradiation at an irradiation intensity of 10 J/cm² for 2 h.

### Cap nucleotides standards

GpppA, GpppG, m^7^GpppA and m^7^GpppG were purchased from New England Biolabs (NEB). NAD, FAD, UDP-Glc, UDP-GlcNAc and dpCoA were purchased from Merck. m^2,2,7^GpppG was purchased from Jena Bioscience. [^13^C_5_]-β-Nicotinamide adenine dinucleotide ammonium salt (^13^C_5_-NAD) and [^13^C_5_]-flavin adenine dinucleotide ammonium salt hydrate (^13^C_5_-FAD) were purchased from Medical Isotopes. [^13^C_6_]-Uridine diphosphate glucose (^13^C_6_-UDP-Glc) disodium salt and uridine diphosphate *N*-acetylglucosamine-^13^C_6_ (^13^C_6_-UDP-GlcNAc) disodium salt were from Omicron Biochemicals. m^7^Gpppm^6^A, m^7^GpppC, m^7^GpppU, Gpppm^6^A, GpppC, GpppU, m^7^GpppNm, GpppNm, ^15^N_5_-m^7^GpppNm, ^15^N_5_-m^7^GpppN, ^15^N_5_-GpppNm and ^15^N_5_-GpppN (N = C, U, G, A or m^6^A) were synthesized as previously described [20, 21] and are now all available from Beijing RNATech.

### Immunofluorescence

Cells were incubated on sterilized slides, fixed in 4% paraformaldehyde for 10 min, permeabilized through cell membranes with 0.2% Triton X-100 for 5 min, and closed with 2% BSA for 1 h. The primary antibody(mouse anti-G3BP1 (Proteintech, 66486-1-IG), rabbit anti-GAPDH (Proteintech, 10494-1-AP), or rabbit anti-eIF4E (Proteintech, 11149-1-AP)) was incubated overnight at 4 °C, the secondary antibody (CoraLite594-conjugated goat anti-mouse and CoraLite488-conjugated goat anti-rabbit) was incubated at room temperature and protected from light for 1 h, and DAPI staining was performed for 5 min; (each addition of new reagents required washing with PBS). After drying, the slices were sealed and observed using a Nikon A1R confocal microscope equipped with 405/488/543/638 nm lasers. Images were captured using the NIS-Elements software, and the objective lenses were selected based on the sample requirements (100×).

### Purification of mammalian SGs

Stressed cells (~ 5 × 10^7^) were collected and lysed with 1 mL of SG lysis buffer(50 mM Tris-HCl pH 7.4, 100 mM KOAc, 2 mM MgOAc, 0.5 mM DTT, 50 µg/mL Heparin, 0.5% NP40, Protease Inhibitor(Thermo Scientific, 78425), and 1 U/µL of RNasin Plus RNase Inhibitor(Promega, N2615)): sonicate at 4 °C for 10 s, lysed on ice for 20 s, and repeated three times [22]; centrifuge at 1000 g for 5 min at 4°C [6] and collect the supernatant containing SG.

### Plasmid transient transfections

The pLVX-AcGFP-G3BP1 plasmid (HonorGene) was transfected into U-2 OS cells by Lipofectamine 2000 Transfection Reagent (Thermo Fisher, 11668030) in conjunction with Opti-MEM Reduced Serum Free Medium. The ratio of plasmid DNA to Lipofectamine was 1: 2.5 for transfection according to the manufacturer’s instructions.

### Purification of mammalian SG cores

SG cores were obtained in AcGFP-G3BP1-U-2 OS cells (~ 5 × 10^8^) with reference to the method of affinity purification of SG cores provided in Khong, et al.[6]. Detailed experimental procedures are provided in the supporting information.

### RNA extraction and mRNA purification

Total RNA was extracted using TRIzol reagent (Thermo Fisher, 15596018CN) and TRIzol LS reagent (Thermo Fisher, 10296010CN) following the manufacturer’s instructions. mRNA was selected from 75 µg total RNA using an mRNA purification kit (Thermofisher, 61006) and purified using. Quality control of total RNA and mRNA was performed using an RNA 6000 Pico kit (Agilent, 5067 − 1513) on a bioanalyzer.

### mRNA hydrolysis

mRNA (85–200 ng) was hydrolyzed using Nuclease P1 enzyme (0.1 U/100 ng mRNA, sigma) in a buffer containing 300 mM sodium acetate (pH 5.5), 10 mM ZnCl_2_, and isotope-labeled standards from a SIL-CN mix. 24 SIL-CNs standards [20]: ^15^N_5_-m^7^GpppNm、^15^N_5_-m^7^GpppN、^15^N_5_-GpppNm、^15^N_5_-GpppN (N = C, U, G, A or m^6^A), ^13^C_5_-NAD, ^13^C_5_-FAD, ^13^C_6_-UDP-Glc, ^13^C_6_-UDP-GlcNAc. The mixture was incubated at 37 °C for 1 h. Sevag (isoamyl alcohol: chloroform = 1: 24) extraction was used to remove the enzyme, and the aqueous phase was separated by centrifugation at 4 °C 10,000 g for 6 min. The supernatant was transferred to a sample vial for HPLC separation and enrichment of CN and related compounds.

#### HPLC

The sample was separated using a quaternary gradient conventional liquid chromatography system (Ultimate 3000, Thermo Fisher Scientific) with an Alltima HP C18 column (4.6 mm × 250 mm, particle size 5 μm, Hichrom) within 1 h. The column temperature was maintained at 25 °C, and the UV detector was set to 260 nm, with a flow rate of 0.8 mL/min. From 0 to 20 min, 100% of mobile phase A (10 mM DBAA in 5% ACN) was used. After 20 min, 1% of mobile phase B (10 mM DBAA in 84% ACN) was added per min for eluting and separating the samples, which were collected in fractions [21]. The fractions were then lyophilized and washed three times with acetonitrile: H_2_O = 3: 7 (v/v) to remove DBAA [20]. The samples were then redissolved in ammonium bicarbonate (NH_4_HCO_3_) solution (pH 7.0) for LC-MS/MS analysis.

#### LC-MS/MS

An ultra-high performance quaternary gradient liquid chromatography system (Vanquish™ Flex, Thermo Fisher Scientific) was coupled to a triple quadrupole mass spectrometer (TSQ Altis, Thermo Fisher Scientific) with an electrospray ionization source operating in both positive and negative modes. Parameters for all 26 unlabeled CN and 24 SIL-CN compounds were set [21]. The sample was separated on a Luna Omega PS C18 column (100 mm × 2.1 mm, 1.6 μm, Phenomenex) at a flow rate of 0.2 mL/min, and the column temperature was maintained at 15 °C. The analytical time was 17 min: 100% solution C (8 mM NH_4_HCO_3_, pH 7) was used from 0 to 5 min, and then 4% solution D (100% methanol) was added every min. Solution D (100% methanol) was used for 0–5 min, then 4% per min [21]. Each CN was identified by HPLC retention time and collision-induced dissociation spectra and quantified using the MRM model and a calibration curve for each CN[20].

#### SiRNA transient transfection

30 nM siRNA (Table S1) was transfected into U-2 OS cells to knock down gene expression using RNATransMate reagent (Sangon Biotech, E607402), following the manufacturer’s instructions. The knockdown effect was validated by western blotting, and changes in mRNA cap structures in knockdown cells were examined. SG assembly was observed by immunofluorescence.

#### Proteomics analysis

Commissioned Shanghai OE Biotech Co., Ltd to perform proteomic quantification using data-independent acquisition (DIA). Detailed experimental procedures are listed in the Supplementary Information. Bioinformatic analysis was performed using the OECloud tools at https://cloud.oebiotech.com/task/. The volcano map (or other graphics) was drawn based on the R (https://www.r-project.org/) on the OECloud platform (https://cloud.oebiotech.com/task/).

## Results

### Mapping mRNA cap epitranscriptome in SGs

Using CapQuant, we defined the landscape of mRNA caps from human osteosarcoma cells (U-2 OS) and SGs formed inside them under several stresses. Focusing first on U-2 OS cells (Supplementary FigureS1−3A), we were able to quantify the components of the cap epitranscriptome. Of the 26 targeted caps, 8 were reproducibly detected for a total of 2417 fmol of caps per µg of mRNA, including 4 metabolite caps and 4 Cap1 cap structures. The structures of the 4 metabolite caps (NAD, FAD, UDP-Glc and UDP-GlcNAc) were unequivocally confirmed by three signature MRM transitions defined with standards (Supplementary FigureS4 A-D). UDP-Glc and NAD being the two most abundant structures is consistent with the relative abundance of these metabolites in human cells [23, 24] and thus with the idea that nucleotide metabolites can initiate transcription [25]. As expected, the 4 Cap1 structures (m^7^GpppNm) comprised the majority of all caps (61.3%, 2099 fmol/µg mRNA) with no cap 0 structures (m^7^GpppN) detected. Consistent with the fact that very few transcriptional start sites (TSS) in humans start with a uridine, m^7^GpppUm comprised only 1% of second-nucleotide subtypes, which ranged from 14 to 25 fmol/µg mRNA. Our analysis further revealed the m^7^Gpppm^6^Am structure proved to be relatively abundant at 40.8% of all mRNA caps (1123 fmol/µg mRNA), this cap demonstrates that 2’-*O*-methylation is not essential in mRNAs, as has been previously suggested to suppress innate host antiviral responses [26].

**Fig. 1:**
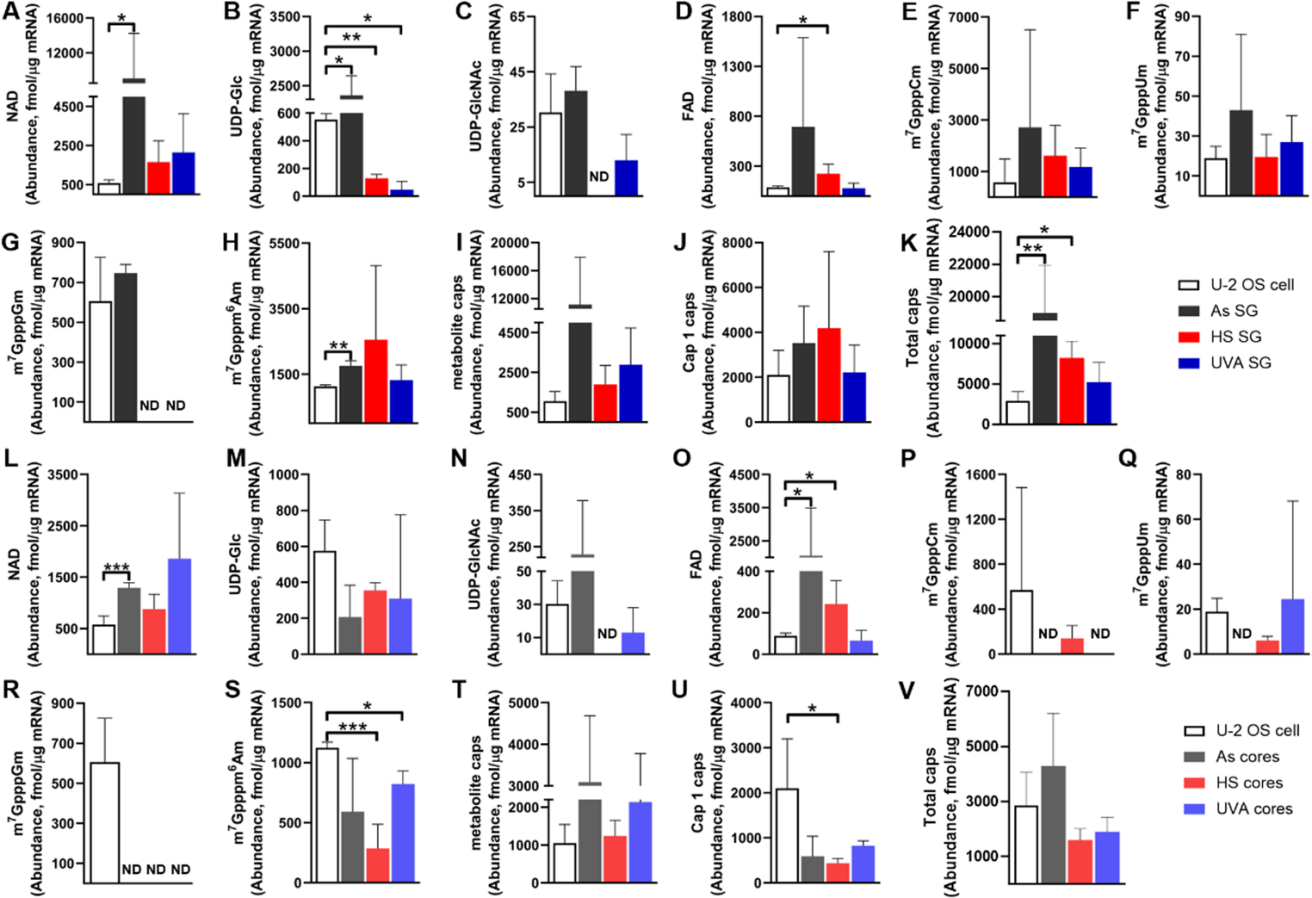
CapQuant quantifies the mRNA cap epitranscriptome in U 2-OS cells with or without stress.** A**, NAD; **B**, UDP-Glc; **C**, UDP-GlcNAc; **D**, FAD; **E**, m^7^GpppCm; **F**, m^7^GpppUm; **G**, m^7^GpppGm; **H**, m^7^Gpppm ^6^Am; **I**: metabolite caps; **J**, Cap1; **K**, total caps, abundance of cap modifications at the SG mRNA level; **L**, NAD; **M**, UDP-Glc; **N**, UDP-GlcNAc; **O**, FAD; **P**, m^7^GpppCm; **Q**, m^7^GpppUm; **R**, m^7^GpppGm; **S**, m^7^Gpppm ^6^Am; **T**: metabolite caps; **U**, Cap1 caps; **V**, total caps; Abundance of mRNA cap modifications in SG cores. Different colors in the graphs indicate different treatment groups, grey As stress; red heat stress; blue UV stress, darker colors represent SGs, lighter colors indicate SG cores, and vertical coordinates indicate the abundance of mRNA caps. Values represent the mean ± SD of three biological replicate experiments. * *p* < 0.05, ** *p* < 0.01, *** *p* < 0.001. ND, not detected

Under stress conditions (arsenite, heat shock, or UV), cytoplasmic proteins represented by G3BP1 aggregate to form SGs (Supplementary Figure S5). The dynamics and composition of SGs are influenced by the cell type, specific stressors and activated signalling pathways [27]. The number and morphology of these SGs show some variation depending on the stressor and treatment conditions [27]. The formation of SGs after stress induction, and the initial particles grow progressively larger through accretion of material and fusion with other stress particles [27]. This suggests that the assembly of mature SGs is a multi-step process that includes an initial nucleation event and an ongoing growth phase. The core structure of these granules can be purified biochemically [6], suggesting that SGs have two distinct layers: a core structure surrounded by a less centralised, and potentially more dynamic, shell structure [28, 29]. Therefore, the study of mRNA cap modifications in SGs in this study was also divided into two parts, one part was to identify the mRNA cap modifications at the level of the overall structure of SGs, and the other part was to study the mRNA cap modifications in them by obtaining SG cores by affinity purification. The types of SG mRNA caps varied considerably in different stress environments (Fig. 1A-K & Table 1), with all 8 caps detected in the As SG; only 6 caps, UDP-GlcNAc and m^7^GpppGm were not detected in the HS SG; and 7 caps were detected in the UV SG, with only m^7^GpppGm not being detected. The mRNA cap content in the samples was calibrated by a calibration curve. Each cap content showed an elevated trend in As SG. In addition to the 2 metabolite caps, UDP-Glc and UDP-GlcNAc, the contents of other caps detected in the HS and UV SGs similarly showed an elevated trend. Notably, the content of the total caps, Cap1’s and metabolite caps was all higher in SGs than in whole cells under all stress conditions tested, indicating enrichment of capped mRNAs in SGs as a general phenomenon occurring when cells are under stress, possibly to prevent certain capped mRNAs from decay or selectively sequestrate certain capped mRNAs. This enrichment of capped mRNAs in SGs is consistent with the congregation of the capping enzyme RNGTT recently reported by Gayen and coworkers [30]. By applying system-level technological tools, we performed a precise quantitative analysis of the RNA cap epitranscriptome. The results show that all Cap1’s and metabolite caps were enriched in SGs, with varying degrees.

**Table 1:** Cap compositions in cellular and SG mRNA

Cap	Level, fmol per µg mRNA (Percentage, %)								
	U-2 OS cell	As SG	HS SG	UVA SG	As SG cores	HS SG cores	UVA SG cores	siRNMT cell	siPCIF1 cell
NAD	576 ± 139 (17.8 ± 13)	9066 ± 4220 (56.3 ± 35.9)	1653 ± 944 (46.4 ± 28.5)	2149 ± 1713 (40.5 ± 22.6)	1291 ± 86 (40.9 ± 18.1)	876 ± 253 (54.4 ± 8.1)	1861 ± 1101 (50.3 ± 32.3)	4304 ± 171 (21 ± 15)	858 ± 186 (4.3 ± 1)
UDP-Glc	554 ± 30 (27.3 ± 11.5)	2359 ± 199 (6.3 ± 6.6)	128 ± 21 (1.9 ± 2.7)	45 ± 43 (0.9 ± 1.4)	207 ± 125 (4.7 ± 3.9)	355 ± 30 (9.4 ± 9.7)	465 ± 380 (6.6 ± 6.4)	112 ± 20 (1.3 ± 0.7)	46 ± 8 (0.3 ± 0.2)
UDP-GlcNAc	30 ± 10 (0.8 ± 1.1)	38 ± 6 (0.1 ± 0.1)	nd	13 ± 8 (0.4 ± 0.5)	229 ± 105 (3.9 ± 2.8)	nd	26 ± 1 (1.2 ± 0.1)	30 ± 10 (2.6 ± 1.3)	59 ± 28 (0.3 ± 0.2)
FAD	89 ± 11 (5 ± 2.9)	693 ± 632 (2 ± 3.4)	223 ± 78 (5.8 ± 4.5)	78 ± 44 (2.2 ± 1.7)	2056 ± 1175 (33.2 ± 12.6)	242 ± 92 (12.6 ± 9)	65 ± 36 (2.8 ± 1.3)	351 ± 297 (3 ± 1.6)	190 ± 64 (1.1 ± 0.7)
m^7^GpppCm	571 ± 744 (12 ±18.1)	2710 ± 2685 (6.4 ± 11.1)	1613 ± 960 (17.7 ± 11)	1174 ± 599 (25.1 ± 17.9)	nd	141 ± 80 (5.4 ± 7.8)	nd	nd	8240 ± 2121 (39.9 ± 6.8)
m^7^GpppUm	19 ± 5 (0.7 ± 0.6)	43 ± 31 (0.4 ± 0.3)	20 ± 10 (0.7 ± 0.6)	27 ± 11 (0.9 ± 0.8)	nd	6 ± 1 (0.4 ± 0.2)	33 ± 40 (2.2 ± 3)	nd	77 ± 11 (0.4 ± 0.1)
m^7^GpppAm	nd	nd	nd	nd	nd	nd	nd	nd	521 ± 117 (2.7 ± 0.9)
m^7^GpppGm	606 ± 155 (8.7 ±9.6)	746 ± 31 (8 ± 11.4)	nd	nd	nd	nd	nd	nd	11,106 ± 1309 (40 ±4.5)
m^7^Gpppm^6^Am	1123 ± 38 (40.8 ± 35.8)	1760 ± 107 (20.4 ± 29.9)	2554 ± 1847 (27.9 ± 18.7)	1316 ± 402 (35.2 ± 10.9)	592 ± 384 (14.4 ± 5.2)	286 ± 174 (17.7 ± 10.7)	822 ± 88 (36.8 ± 7.7)	7310 ± 2310 (71.1 ± 11)	7310 ± 2310 (41 ± 22.8)
Total caps	2417 ± 1137	10,985 ± 8148	5031 ± 3396	2223 ± 1047	4284 ± 1568	1599 ± 352	2801 ± 1599	10,901 ± 4382	22,015 ± 8074

Values (expressed as femtomoles per microgram of mRNA or as a percentage of each detected cap structure) represent the mean ± standard deviation of three technical replicates of three independent cultures of all cell lines, SGs and SG cores after three independent treatments of the cells under different conditions, and cells after transfection with siRNA knockdown. nd, not detected

We purified U-2 OS cell SG cores using protein affinity purification (Figure S5) and cap analysis results revealed that the composition and abundance of mRNA 5’ caps in the cell SGs and SG cores differ significantly under different stress conditions. In SG cores, relatively few types of caps were detected (Fig. 1L-V & Table 1). The NAD cap showed an increasing trend in SG cores formed under all three stress conditions (Fig. 1L); the FAD cap showed a significant increasing trend in As and HS cores (Fig. 1O); the UDP-Glc and m^7^Gpppm^6^Am cap content showed a decreasing trend, and the content of the mRNA m^7^Gpppm^6^Am cap in the HS and UVA SG cores was significantly lower than that of the cells (Fig. 1S). Except for a slight increase in abundance of total caps in As cores compared with that in cells, there was a decreasing trend in both HS and UVA cores (Fig. 1V), especially in HS cores, where the content of mRNA Cap1 was significantly lower than that in cells (Fig. 1U).

### RNMT regulates mRNA cap modification and affects SG assembly

**Fig. 2:**
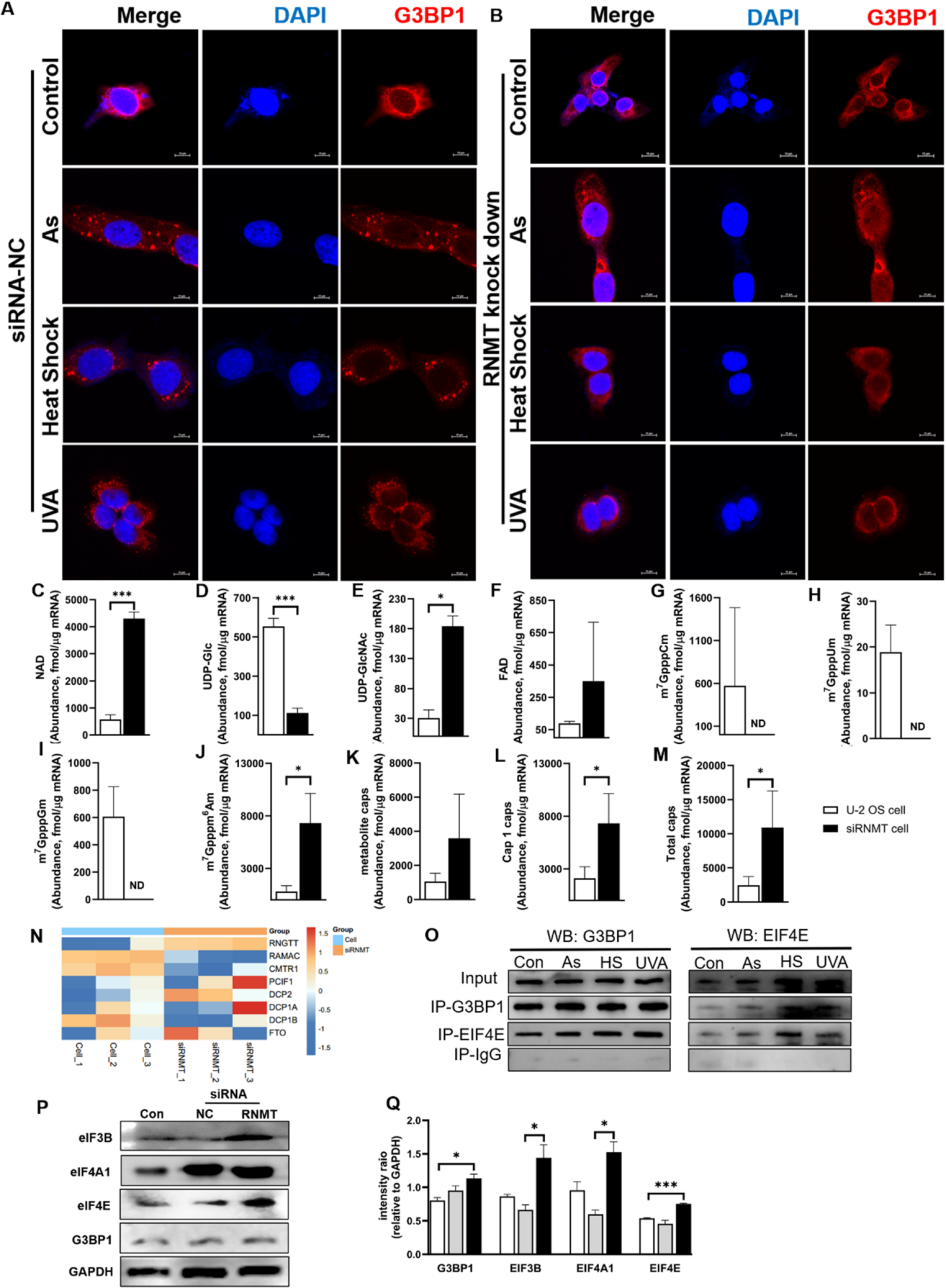
Changes in the content and composition of cellular mRNA caps and stress response after knockdown of RNMT expression. **A**: Confocal immunofluorescence analysis of U-2 OS cells treated with NC-siRNA transfected by stress treatment using G3BP1 protein (red); **B**, Confocal immunofluorescence analysis using G3BP1 protein (red labeled) stress-treated RNMT knockdown cells, (The scale in **A & B** is 10 μm); **C**, NAD; **D**, UDP-Glc; **E**, UDP-GlcNAc; **F**, FAD; **G**, m^7^GpppCm; **H**, m^7^GpppUm; **I**, m^7^GpppGm; **J**: m^7^Gpppm ^6^Am; **K**, metabolite caps; **L**, Cap1 caps; **M**, total caps, CapQuant detects changes in cap modification at the mRNA level in cells after RNMT knock down, (* *p* < 0.05, ** *p* < 0.01, *** *p* < 0.001; ND stands for Not Detected); **N**, Proteomic analysis of intracellular cap modifying enzyme gene expression before and after knockdown of RNMT; **O**, Co-IP detection of G3BP1 and eIF4E protein interactions; **P**, Western blot (WB) analysis of G3BP1 and cap binding proteins eIF3B, eIF4A1 and eIF4E; **Q**, Quantification of protein bands using ImageJ software

RNMT serves as the core enzyme of the mRNA capping reaction [31]. In order to investigate whether mRNA cap is involved in the cellular stress response, the present study used siRNA technology to specifically knock down RNMT in cells (FigureS6) and carried out simultaneous stress observation and end-cap assay experiments, aiming at revealing the potential connection between mRNA cap and SG. In the experiments, U-2 OS cells without any treatment (Con) as well as cells transfected with the same concentration of siNC-RNA (siNC cell) were used as control groups to carry out the study together with siRNMT cells. Observed with the aid of laser confocal microscopy, under the same stress conditions, siNC cells (Fig. 2A) were also successful in forming SGs, whereas in siRNMT cells (Fig. 2B), no obvious SG structure could be observed. This significant difference prompted us to further explore in depth the changes that occur in intracellular SG assembly as a result of RNMT knockdown.

To explore the link between this phenomenon and the changes in mRNA cap modifications and related proteins caused by RNMT knockdown, we aim to gain an initial understanding of the mechanism by which the cap structure impacts SG formation. First, as expected, knockdown of RNMT caused widespread changes in the level of intracellular mRNA modifications. Specifically 3 Cap1 structures (Fig. 2G-I & Table 1), m^7^GpppCm, m^7^GpppUm, and m^7^GpppGm, became undetectable after the knockdown. Notably, m^7^Gpppm^6^Am cap levels significantly increased, suggesting it follows a distinct pathway (Fig. 2J). Moreover, among the four metabolite caps, the level of UDP-Glc was significantly decreased (Fig. 2D), while the levels of both NAD and UDP-GlcNAc caps showed a significant increase (Fig. 2C E). This finding suggests that the expression level of RNMT may similarly influence the homeostasis of non-canonical caps.

Further proteomic analysis revealed that the down-regulation of RNMT expression not only affected specific cap structures, but also induced changes in the overall levels of intracellular cap modifying enzymes, including capping and decapping enzymes, e.g. elevated expression of the cap-specific adenosine methyltransferase PCIF1 was associated with a significant decrease in the FTO demethylase (Fig. 2N). These changes in end cap modifying enzymes may be one of the reasons for the overall changes in end cap modification in the cell. This suggests that RNMT plays a central role in regulating the mRNA cap modification network, and that changes in its expression level may trigger a series of complex molecular responses that impact on intracellular cap modification homeostasis.

To our best knowledge, there is no study reporting RNMT’s direct role in SG formation. Yet, RNMT knockdown prevents SG formation under stress. This suggests cap structures catalyzed by RNMT and associated proteins may be crucial for SG formation. We hypothesize that reduced Cap1 may impede translation of key SG proteins or that Cap1 itself connects SG proteins and RNA, causing RNA aggregation instead of degradation. G3BP1 co-localization and functional linkage with the cap-binding protein, eIF4E, has been widely confirmed by many studies [32, 33]. To test this, we first examined interactions between the SG key protein G3BP1 and eIF4E, which could recruit capped RNA to SGs. Co-immunoprecipitation (Co-IP) results confirmed the interaction between G3BP1 and eIF4E in the cells with or without stress and indicated that the interaction was strengthened upon all stress conditions tested (Fig. 2O), suggesting that their interaction may play a role in the formation of stress granules. With the aid of laser confocal microscopy, we observed that eIF4E was involved in the assembly process of SGs under different stress conditions (FigureS7). Under stress conditions, both G3BP1 and eIF4E were found to be localized in stress granules, which further supports their collaborative role in the formation of stress granules.

However, Western Blot assay showed that the expression levels of G3BP1, the main regulatory protein of SGs, as well as some of the cap-binding proteins (eIF3B, eIF4A1, eIF4E) were significantly up-regulated after RNMT knockdown (*p* < 0.05), with the up-regulation of the expression of the cap-binding protein, eIF4E, being particularly significant (*p* < 0.001) (Fig. 2P **& Q**). In RNMT knockdown cells, levels of G3BP1 and the cap-binding protein eIF4E rised, yet stress granules did not form under stress. This suggests caps, not these proteins, are key to stress granule formation.

### PCIF1 knockdown changes mRNA cap modifications but not SG assembly

We observed that SGs failed to form in U-2 OS cells after knockdown of RNMT. Consistent with literature findings, the level of several Cap1’s decreases obviously. However, to our surprise, the m^7^Gpppm^6^Am cap level increases substantially. It has been noted that m^6^Am modification on RNA can influence intracellular SG assembly [18].

**Fig. 3:**
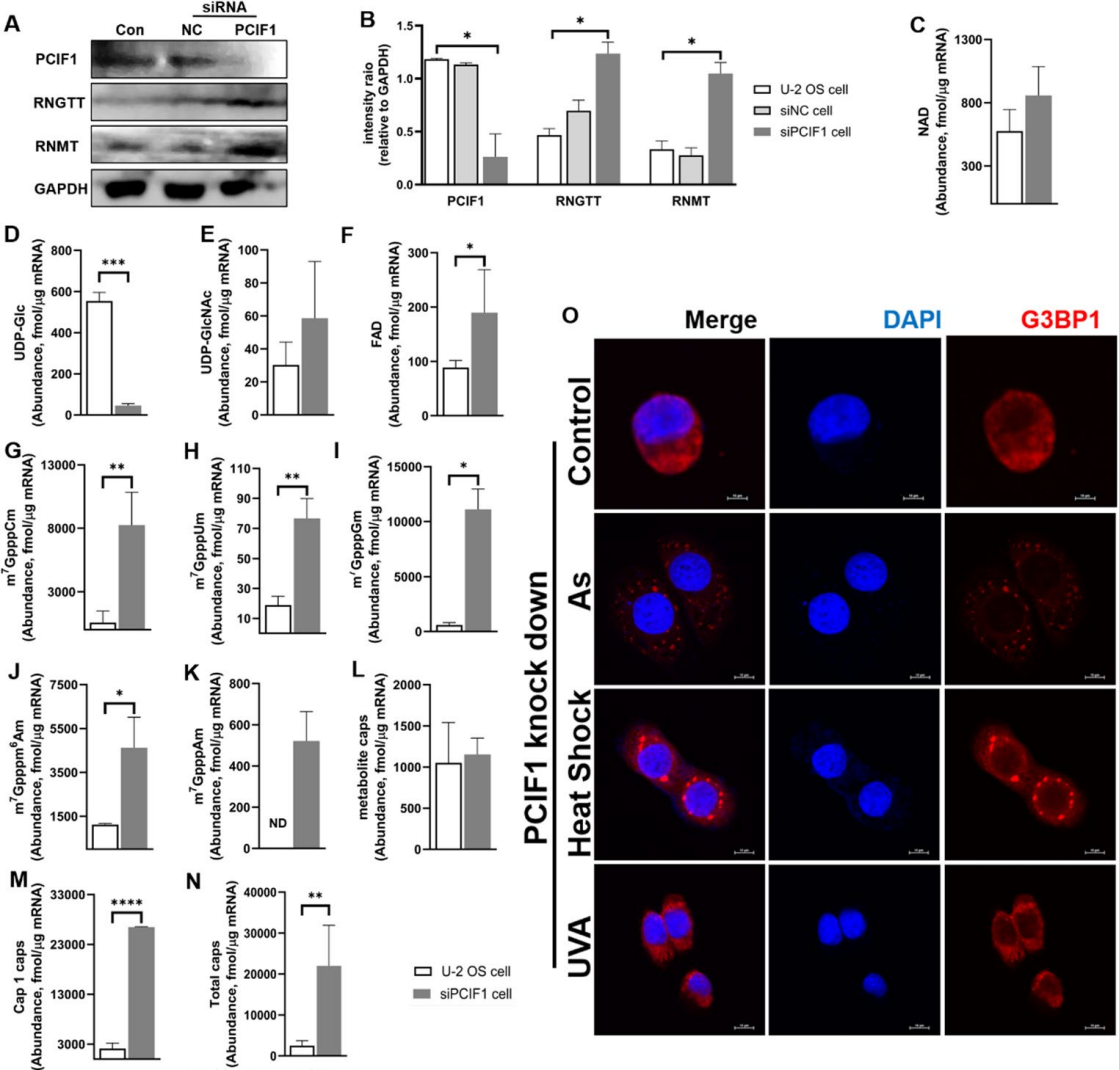
Knockdown of the cap-modifying enzyme PCIF1 led to changes the content and composition of mRNA caps in U-2 OS cells.** A:** Western blot for protein expression levels of PCIF1, RNGTT and RNMT; **B**: Quantification of protein bands using ImageJ software; **C**, NAD; **D**, UDP-Glc; **E**, UDP-GlcNAc; **F**, FAD; **G**, m^7^GpppCm; **H**, m^7^GpppUm; **I**, m^7^GpppGm; **J**, m^7^Gpppm ^6^Am; **K**: metabolite caps; **L**, Cap1 caps; **M**, total caps, CapQuant detects changes in caps in mRNA in cells after PCIF1 knock-down; **O**: Confocal immunofluorescence analysis using G3BP1 protein(red labeled) stress-treated PCIF1 knockdown cells. (* *p* < 0.05, ** *p* < 0.01, *** *p* < 0.001; The scale in O is 10 μm)

The PCIF1, an mRNA 2’-*O*-methyladenosine-N(6)-methyltransferase, is responsible for the formation of cap-adjacent N6,2’-*O*-dimethyladenosine (m^6^Am) by methylating the adenosine at the first transcriptional position of the capped mRNA [34]. This enzyme is highly conserved in mammalian cells and directly affecting the abundance of m^6^Am modification at the RNA level within the cell [35]. Based on these, in order to further dissect the association between the increased level of m^7^Gpppm^6 ^Am and SG assembly in RNMT knockdown cells, we knocked down the phosphorylated PCIF1 (Fig. 3A-B). We analyzed the subsequent changes in cap modifications within the cells (Fig. 3C-N **&** Table 1). In contrast, when PCIF1 is knocked down, several changes are observed: the detection of m^7^GpppAm cap, which was previously undetectable, an increase in m^7^Gpppm^6^ Am cap levels, and a rise in Cap1 (m^7^GpppCm, m^7^GpppUm, m^7^GpppGm and m^7^Gpppm^6^ Am). Comparing the changes in intracellular metabolite cap modification levels after RNMT knockdown, we observed a consistent trend: a significant decrease in the cap modification abundance of UDP-Glc (Fig. 3D) and an increase in the other 3 metabolite caps (Fig. 3C E & F). Notably, SGs do form under these conditions (Fig. 3O). This indicates that the presence of m^7^GpppAm cap, in conjunction with elevated m^7^Gpppm^6^ Am cap levels and the restoration of Cap1, may create a cap modification profile that is conducive to SG assembly.

### Proteomic analysis of RNMT knockdown cells and SG proteins

The mRNA cap has an important regulatory role in the intracellular translation of mRNAs into proteins, the changes in intracellular cap modification caused by RNMT knockdown may affect the translation efficiency and stability of mRNAs, which in turn affects intracellular protein levels. Therefore, studying the overall effect of intracellular cap modification changes induced by RNMT knockdown on the protein level is important for further understanding the mechanism by which intracellular RNMT knockdown affects SG assembly. We performed an in-depth proteomic analysis of U-2 OS cells and the three SGs within them before and after knockdown of RNMT by DIA proteomic quantification. In this study, we identified a total of 9397 proteins in normal cells. By Principal Component Analysis (PCA) analysis, we found that the protein levels in cells or intracellular after RNMT knockdown were significantly different from those in normal cells (Figure S8) with very high correlation in experimental replicates, which suggests that knockdown of RNMT has a broad effect on the cellular proteome.

**Fig. 4:**
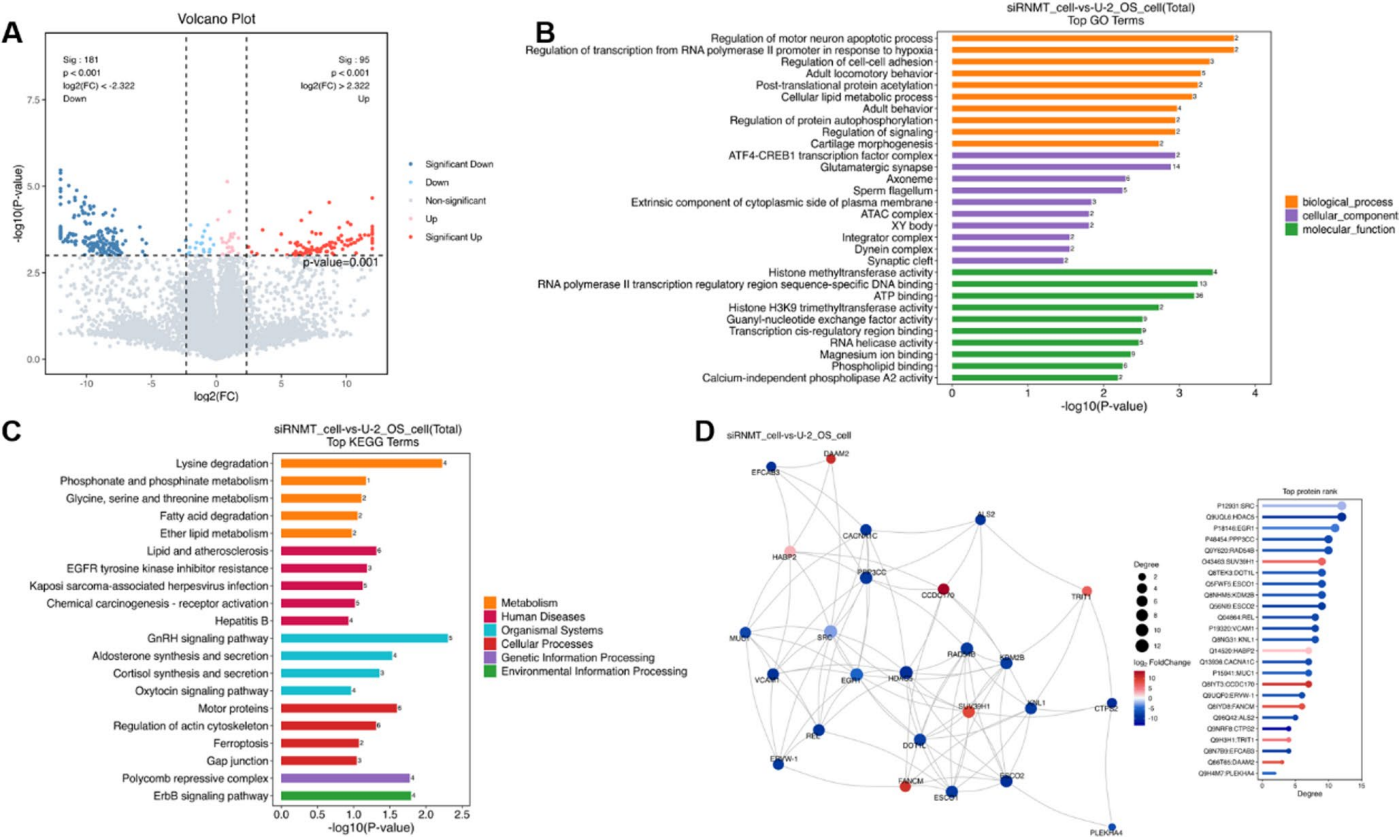
Proteomic analysis of siRNMT cells.** A**: Proteomics test PCA analysis DEP; **B**: Top 30 pathways for DEP GO enrichment in proteomics; **C**: DEP ratio analysis of KEGG pathway enrichment of the first 20 up-regulated; **D**: Proteomics DEP differential protein interactions (Top25 connectivity)

The differential screening condition was set as Fold Change > 5, P-value < 0.001 to assess the differential protein expression between groups. In RNMT knockdown cells (siRNMT), we identified 276 differentially expressed proteins (DEPs), including 95 up-regulated and 181 down-regulated proteins (Fig. 4A). Gene Ontology (GO) analysis revealed that these differentially expressed proteins were mainly involved in key biological processes such as apoptosis regulation, metabolic reprogramming (including lipid and amino acid metabolism) and motor behavior, and were significantly enriched in synaptic structures, motor cilia and chromatin complexes (Fig. 4B). Kyoto Encyclopedia of Genes and Genomes (KEGG) pathway analysis further revealed significant alterations in metabolic pathways (e.g., lysine degradation), cancer-related pathways (e.g., EGFR inhibitor resistance), and cellular processes (e.g., iron death) (Fig. 4C). Protein-protein interaction (PPI) network analysis (top 25) identified 4 key functional modules: the HABP family of proteins as core hubs interacting with ECM receptors and integrins; histone modifying enzymes in complex with ATAC forming an epigenetic regulatory network; the metabolic module in which ACSL4 is connected to AASS via ATP6V1A; and signaling proteins encompassing the EGFR/ErbB pathway with cytoskeletal proteins module. The study also identified TP53 and MYC as key central proteins to regulate apoptosis and metabolism-related factors, respectively, as well as the important role of HSP90AA1 in protein quality control (Fig. 4D).

Under identical differential screening conditions, the proteomes of three types of SGs were analyzed. Compared to the U-2 OS proteome (FigureS9), the As SG group exhibited 644 significantly DEPs (411 downregulated, 233 upregulated). The HS SG group showed 424 significant changes (203 upregulated, 221 downregulated), a notably lower number than the As SG group, indicating a stress-type-specific response. The UVA SG group displayed 655 significantly altered proteins (421 downregulated, 234 upregulated), with volcano plots revealing clear separation in both fold change and statistical significance dimensions. In the GO analysis, the DEPs from different SGs were primarily associated with essential intracellular physiological processes in the biological process category (Figure S10). The cellular component terms were predominantly enriched in organelle-related structures, while molecular functions were largely linked to critical cellular activities, reflecting tight regulation of vital structures and functions under stress. KEGG pathway analysis revealed significant involvement of metabolic pathways across all SG types: As SGs were enriched in metabolic disturbance-related pathways, HS SGs in metabolic reprogramming pathways, and UVA SGs in endocrine regulation pathways (Figure S11). These findings underscore the pivotal role of metabolic adaptation in cellular stress responses. PPI network analysis (top 25) revealed that DEPs across all SG types were implicated in cellular metabolic regulation (FigureS12). However, distinct functional preferences were observed: As SG DEPs were predominantly enriched in mitochondrial functions, HS SG DEPs were biased toward epigenetic regulation, while UVA SG DEPs potentially participated in photodamage repair processes.

**Fig. 5:**
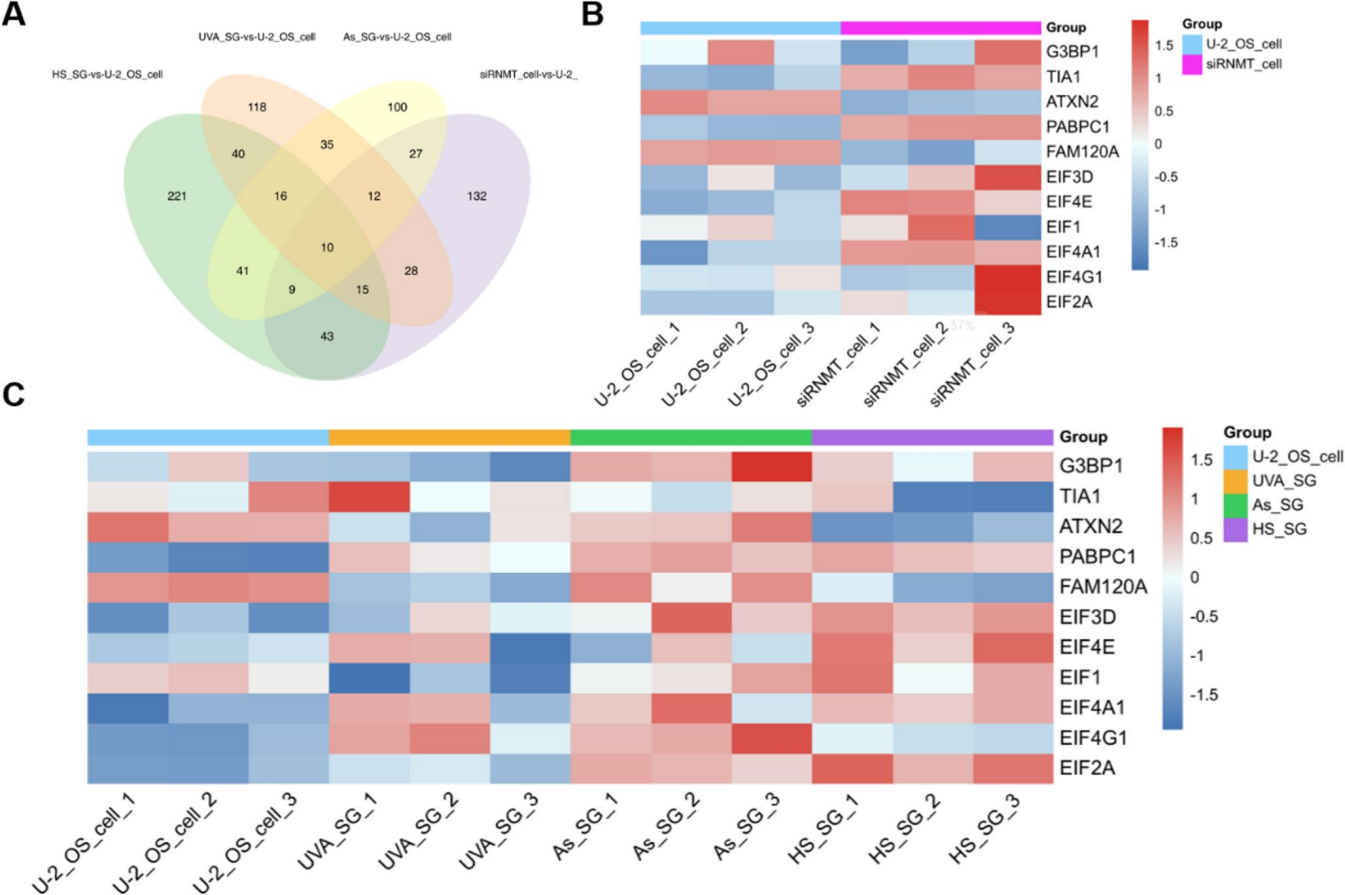
DEPs analysis of siRNMT cells and SGs. **A**: Venn analysis of DEPs reveals shared and condition-specific signatures across experimental groups; **B**: Analysis of differential expression of SG regulatory proteins and cap-binding proteins after RNMT knockdown; **C**: Differential expression of SG regulatory proteins and cap-binding proteins in SGs

In an in-depth analysis of the changes in cellular protein levels due to RNMT downregulation and its effect on SG assembly, we compared DEPs that were upregulated/downregulated in SG and siRNMT. While 129 proteins showed significantly differential expression in three SG types (FigureS13), only 7.8% (10/129) were shared with siRNMT cells (Fig. 5A), suggesting that RNMT may regulate SG formation primarily through cap modification-dependent pathways (e.g., mRNP assembly or mRNA sorting) rather than large-scale proteome remodeling. And no direct correlation with cellular stress response has been reported among these 10 shared DEPs so far. Targeted evaluation of known SG regulators and translational initiation factors. In siRNMT, the reported major regulatory proteins of SG formation (e.g., G3BP1[36]), SG backbone proteins [37] (e.g., PABPC1, ATXN2), and cap-binding proteins (e.g., eIF4E) did not show significant changes under stress conditions (Fig. 5B). Our analysis revealed that the translation levels of these proteins remained similar to their enrichment in SGs, as observed in both RNMT knockdown and control cells (Fig. 5C). Proteomic analysis of these knockout cells and SGs revealed that the translation levels of these proteins are not the primary factor impacting intracellular SG assembly after RNMT knockdown.

## Discussion

SG proteins are widely regarded as the main regulators of SG assembly [7, 38, 39]. In recent years, with the deepening of SG research, researchers have found that RNA, including its modifications, plays an important regulatory role in the formation of intracellular SGs [40, 41]. RNA modifications, such as m^6^A modifications, act as “switches” with in RNA structure influencing RNA interactions with RNA-binding proteins [42]. While mRNAs are known to be integral components of SG RNAs [6], the specific role of mRNA cap modifications in intracellular stress responses has not been extensively reported. CapQuant represents a cutting-edge method for the precise and sensitive quantification of the epitranscriptome of RNA caps in various organisms [20]. Using CapQuant technology, we quantitatively analyzed mRNA cap modifications during cellular stress responses and found significant differences in both RNAs and proteins involved in stress responses across various SGs, aligning with literature reports [8, 43, 44]. This finding suggests that SGs exist at two distinct structural levels, with mRNAs involved in SG assembly presenting at two different cap modification levels.

RNMT not only directly participates in the formation of the cap structure through its catalytic activity [45], but also regulating its function through interactions with other proteins and post-transcriptional modifications, which are essential for eukaryotic cell growth and virus survival [46]. Our findings revealed that after knockdown of RNMT expression, no obvious SG structures could be observed in cells after knockdown of RNMT expression, sparking a great interest in the link between RNMT and cellular stress response. We analyzed why intracellular RNMT knockdown may affect the assembly of cellular SGs after RNMT knockdown at the mRNA cap modification and protein levels, respectively. In knockdown RNMT cells, the overall cap modification levels were changed. Notably, the downregulation of RNMT resulted in the loss of 3 caps (m^7^GpppCm, m^7^GpppUm, and m^7^GpppGm) in Cap1; however, a significant increase in the abundance of the m^7^Gpppm^6^Am modification, which also serves as a Cap1, may be attributed to the upregulated expression of the cellular m^6^Am-specific cap modifying enzyme PCIF1. Further analysis is required to elucidate the mechanism for the compromised assembly of intracellular SGs after RNMT knockdown.

Surprisingly, the level of the m^7^Gpppm^6^ Am cap was substantially increased in siRNMT cells. Therefore, we undertook to explore the potential function of the m^7^Gpppm^6^ Am cap in SG formation. To our surprise, the level of the m^7^Gpppm^6^Am cap and the other four Cap1’s all went up significantly in siPCIF1 cells. To understand this observation, we examined the protein expression levels of PCIF1, RNGTT and RNMT in siPCIF1 and control cells and the results revealed downregulation of PCIF1 and upregulation of RNGTT and RNMT. The upregulation of RNGTT and RNMT may serve as a compensatory mechanism to maintain efficient translation in siPCIF1 cells lacking PCIF1 and the resulting increase in the m^7^GpppAm cap could lead to increased m^7^Gpppm^6^Am cap via PCIF1 catalysis, albeit probably at a lower rate in the knockdown cells.

Our results showed that the levels of metabolite caps in siPCIF1 cells changed in a similar way to those in siRNMT cells, suggesting that the biogenesis and metabolism of metabolite caps remained largely unaffected when the biogenesis and metabolism of m^7^G-caps in the cells was altered caused by genetic knockdown of RNMT or PCIF1. Moreover, the metabolite caps may have distinct biological functions and be involved in various metabolic pathways. For instance, TIR structural domain proteins that can specifically act on NAD-RNA and possess decapping activity [47, 48]. However, the regulatory pathways of metabolite caps are not well-defined. Interestingly, SG structures could still be observed after stress treatment in siPCIF1 cells, which suggests that the changes in metabolite cap levels and elevated m^7^Gpppm^6^ Am cap content observed in siRNMT cells are not the primary cause of the observed effect on SG assembly. We speculate that in the later stages of the stress response, cells more precisely regulate gene expression through Cap1 to adapt to sustained stress.

We explored the effects of RNMT knockdown on intracellular protein expression using proteomics analysis. Our findings revealed that RNMT knockdown not only altered the levels of cap modifications in cells, but also significantly affected protein expression. In siRNMT cells, we observed significant differences in protein expression levels compared to control cells. Although knockdown of RNMT did not have a significant effect on the expression of some of the widely studied major regulatory proteins in SGs, or these proteins showed similar expression trends in SGs, we found that proteins that were significantly differentially expressed in siRNMT cells were not directly associated functionally and in terms of regulatory pathways with the three SGs in our study. Furthermore, some DEPs showed similar expression patterns in siRNMT cells. Currently, there is no direct evidence that these proteins play a role in the cellular stress response, and their potential connection with SGs requires further investigation.

The effects of RNMT knockdown on cells are multifaceted, and our proteomic analysis highlight an important role for RNMT in regulating cellular protein expression. Despite RNMT knockdown has limited effects on the major known regulatory proteins in the SG, we determined that RNMT has an impact on SG formation and function. There may be other uncharacterized proteins or regulatory mechanisms that play a key role in SG formation and dynamics in the context of RNMT knockdown. These proteins may be involved in the regulation of the cellular stress response or related to the formation and disassembly of SGs. Future studies can focus on the functional characterization of these proteins and their mechanisms of action in the cellular stress response, leading to a more comprehensive understanding of the effects of RNMT knockdown on the cellular stress response.

In summary, our results underscore the differential enrichment of mRNA caps in SGs under various stress conditions. While knockdown of RNMT and PCIF1 both substantially changed the content and composition of mRNA caps, knockdown of RNMT, but not PCIF1, led to failure of SG formation under different stress conditions. Notably, proteomic, Co-IP and confocal immunofluorescence analysis revealed that cap-protein interactions contribute to the partitioning of mRNAs into SGs. Furthermore, our results suggest the mRNA cap as an important recognition element of mRNAs essential for the assembly of intracellular SGs. The findings revealed by this study provide new perspectives for understanding the biogenesis, regulation and function of SGs, and provide new directions for future research.

## Supplementary Information

Below is the link to the electronic supplementary material.

Supplementary Material 1 (DOCX 7.28 MB)

## Data Availability

All data are available in the main text or supplementary materials and the raw mass spectrometry data have been deposited to the ProteomeXchange Consortium via PRIDE.
