# Supplementary material for: mRNA caps accumulate in stress granules and are essential for their formation
Source: Cell Mol Life Sci. 2025 Nov 25;82(1):420. doi: 10.1007/s00018-025-05947-8 (PMC12647448; doi:10.1007/s00018-025-05947-8)
Supplement: Supplementary file 1 — Supplementary Material 1 (DOCX 7.28 MB) [file 18_2025_5947_MOESM1_ESM.docx]

**Supplementary Materials**

**mRNA Caps Accumulate in Stress Granules and Are Essential for Stress-Induced Formation**

Mingxin Guo^1^, Ying Feng^1^, Guantong Qi^1^, Wenran Ma^1^, Xiaoli He^1^, Ruiqi Li^1^, Yu Liu^1✉^, Jin Wang^1✉^

Supplemental experimental procedures

Table S1 siRNA sequences

Figure S1 QC analysis of Total RNA on the RNA Pico chip of the Agilent 2100 Bioanalyzer

Figure S2 QC analysis of mRNA on an Agilent 2100 Bioanalyzer RNA Pico chip

Figure S3 HPLC trace for the separation of the enzymatic digestion mixture of mRNA

Figure S4 Analysis of 5′ cap structures in mRNA by CapQuant

Figure S5 Confocal immunofluorescence analysis of untreated or poststress-treated U-2 OS cells (housekeeping gene glyceraldehyde-3-phosphate dehydrogenase GAPDH labeled with Green) was performed using the G3BP1 protein (RED-labeled)

Figure S6 Confocal immunofluorescence analysis of untreated or post-stress-treated U-2 OS cells using G3BP1 protein (RED-labeled) (cap-binding protein eIF4E labeled with Green)

Figure S7 Confocal immunofluorescence analysis of U-2 OS cells treated with NC-siRNA transfected by stress treatment using G3BP1 protein (red-tagged)

Figure S8 Proteomics test PCA analysis DEP

Figure S9 Top 30 pathways for DEP GO enrichment in proteomics

Figure S10 DEP ratio analysis of KEGG pathway enrichment of the first 20 up-regulated

Figure S11 20 pathways down-regulated by proteomics DEP KEGG enrichment

Figure S12 Proteomics DEP differential protein interactions (Top25 connectivity)

**Supplemental experimental procedures**

**Purification of mammalian SG cores**

AcGFP-G3BP1-U-2 OS cells (~5 × 10^8^) were collected and lysed with 1 mL of SG lysis buffer: sonicate at 4°C for 10 seconds, lysed on ice for 20 seconds, and repeated three times; centrifuge at 1000 g for 5 min at 4°C and collect the supernatant containing SG.

Enrichment and purification of mammalian SG cores from supernatants was first performed by collecting SG cores by high-speed centrifugation of the SG supernatant, removing the supernatant and resuspending the precipitate with SG lysis buffer, and repeating the process to further enrich SG cores. Next, the precipitate was adjusted by low-speed centrifugation and the supernatant (enriched for SG cores) was transferred to a new tube. The enriched fractions were then pre-washed twice using DEPC-treated Protein A Dynabeads to remove non-specific binding substances. Anti-GFP antibody was added and incubated to specifically bind the GFP-labeled SG core. After removing the unbound antibody by centrifugation, the precipitate was resuspended again with lysis buffer and magnetic beads and incubated for binding, so that the antibody binds to the magnetic beads to achieve specific capture and purification of the SG core, and pure SG cores samples were obtained, and SG cores RNA and proteins were extracted by TRIzol LS reagent.

**Proteomics analysis**

Protein concentration measurement: BCA protein concentration assay was used to determine the protein concentration in the samples.

Trypsin digestion: Based on the measured protein concentration, appropriate amount of protein was taken and adjusted to the same concentration and volume with lysis solution. Next, DTT was added to the protein solution for reduction reaction, and after cooling, iodoacetamide was added for alkylation reaction. After that, the protein was precipitated with acetone, and the precipitate was collected by centrifugation and re-solubilized with NH_4_HCO_3_. Finally, trypsin Trypsin-TPCK was added for overnight digestion and the enzymatic reaction was terminated by pH adjustment.

Peptide desalting: The enzymatic peptides were subjected to a process of desalting using SOLATM SPE 96-well plates.

LC-MS/MS High Resolution Mass Spectrometry Detection: High-resolution mass spectrometry was performed using LC-MS/MS technology. Firstly, before mass spectrometry injection, the iRT standard was mixed with the sample to be tested at the ratio of 1:20, which was used as an internal standard for the calibration of the chromatographic system and quantitative quality control. Next, a C18 analytical column was used for peptide separation, which ensured effective separation of peptides through a carefully designed elution gradient and mobile phase composition. Mass spectrometry analysis was then performed on a timsTOF HT mass spectrometer with DIA mode for data acquisition. Mass spectrometry parameters such as electrospray voltage, capillary temperature, drying gas flow rate, mass range, collision energy, and ion mobility range were optimized to achieve high-resolution and high-sensitivity detection.

Data analysis: The process of database searching and quantitative analysis of protein DIA was completed by merging mass spectrometry data through PASER software. The sequence file of the search library used for the analysis was uniprot-Homo sapiens-9606-2024.2.1.fasta.

Table S1 siRNA sequences

| siRNA | Accession Number | sequence information (5′-3′) |
| --- | --- | --- |
| NC-siRNA | -- | S: UUCUCCGAACGUGUCACGUTT  A: ACGUGACACGUUCGGAGAATT |
| RNMT-siRNA | NM_003799.3 | S: CCUGCAAAUGAGAGUUCUAAATT  A: UUUAGAACUCUCAUUUGCAGGTT |
| PCIF1-siRNA | NM_022104.4 | S: GUCCCUACUACUUCAACCGAUTT  A: AUCGGUUGAAGUAGUAGGGACTT |

(S: Sense; A: Antisense)


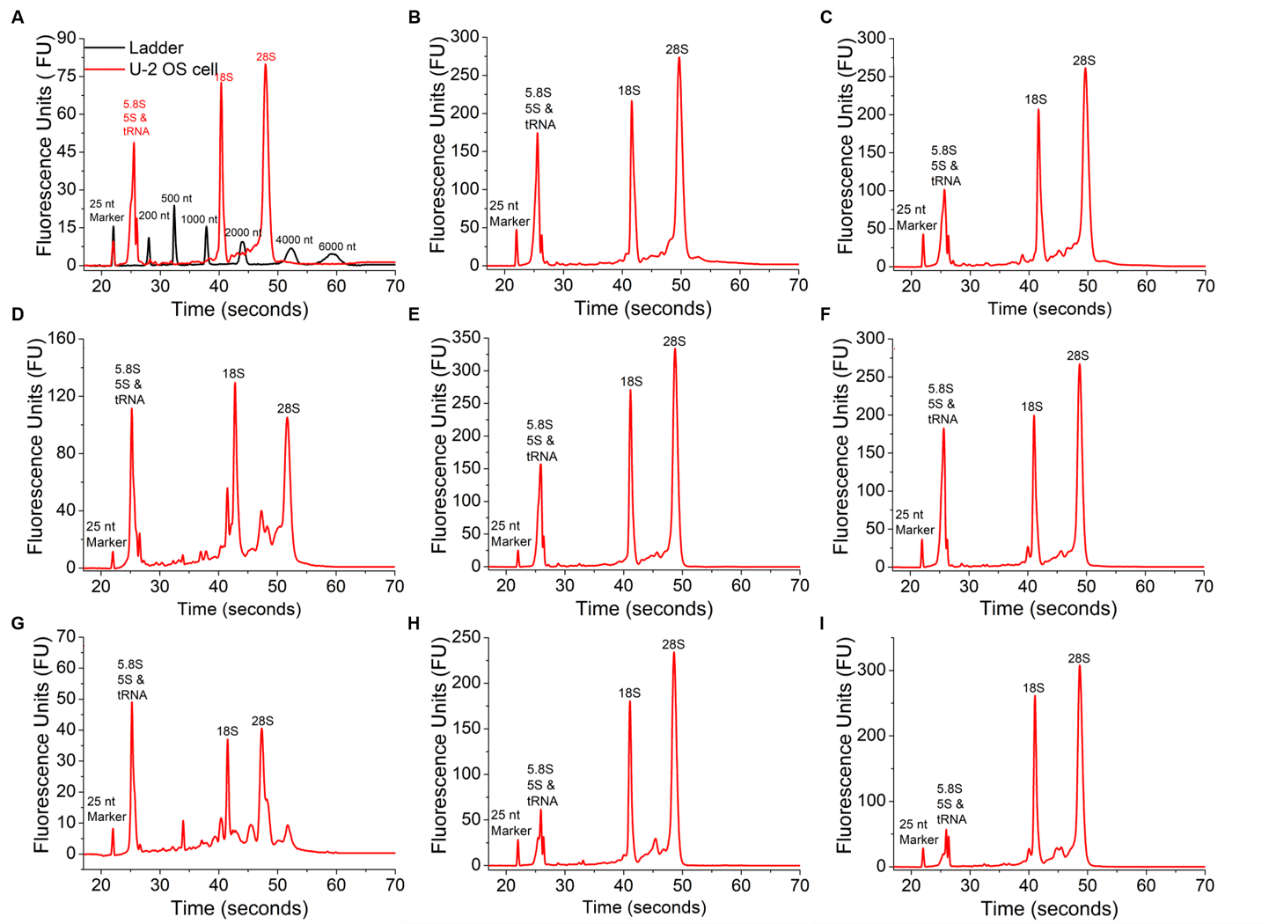


**Figure S1 QC analysis of Total RNA on the RNA Pico chip of the Agilent 2100 Bioanalyzer.** A: U-2 OS cell (total RNA labeled in RED, ladder labeled in BLACK); B: cap-modifying enzyme RNMT Knockdown cell; C: modifying enzyme PCIF1 Knockdown cell; D: NaAsO_2_ Stress Granules (As SG); E: Heat shock Stress Granules (HS SG); F: UVA Stress Granules (UVA SG); G: NaAsO_2_ Stress Granule cores (As SG cores) ; H: Heat shock Stress Granule cores (HS SG cores); I: UVA Stress Granule cores (UVA SG cores).


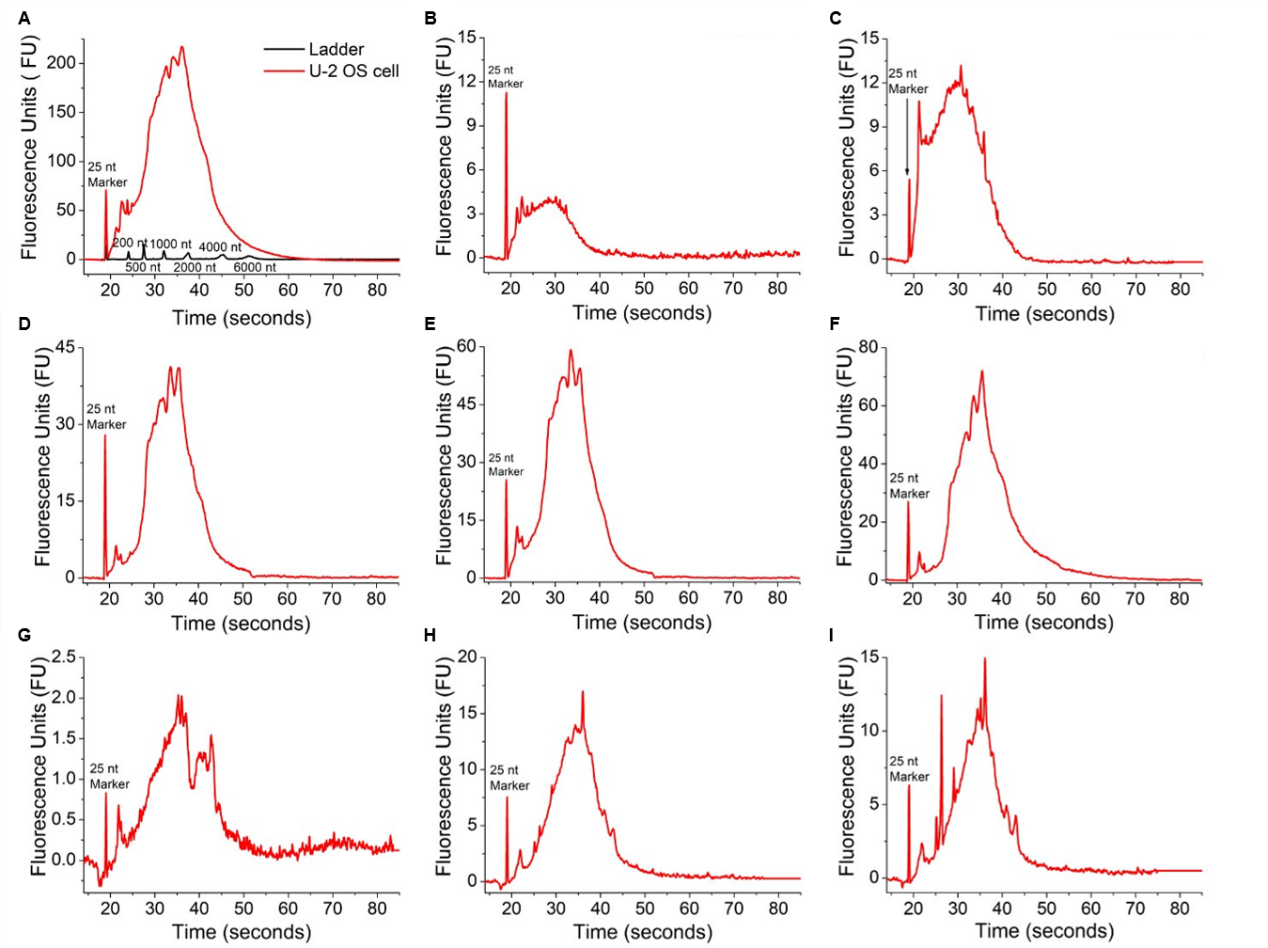


**Figure S2** **QC analysis of mRNA on an Agilent 2100 Bioanalyzer RNA Pico chip.** A: U-2 OS cell (mRNA labeled in RED, ladder labeled in BLACK); B: cap-modifying enzyme RNMT Knockdown cell; C: modifying enzyme PCIF1 Knockdown cell; D: NaAsO_2_ Stress Granules (As SG); E: Heat shock Stress Granules (HS SG); F: UVA Stress Granules (UVA SG); G: NaAsO_2_ Stress Granule cores (As SG cores) ; H: Heat shock Stress Granule cores (HS SG cores); I: UVA Stress Granule cores (UVA SG cores).


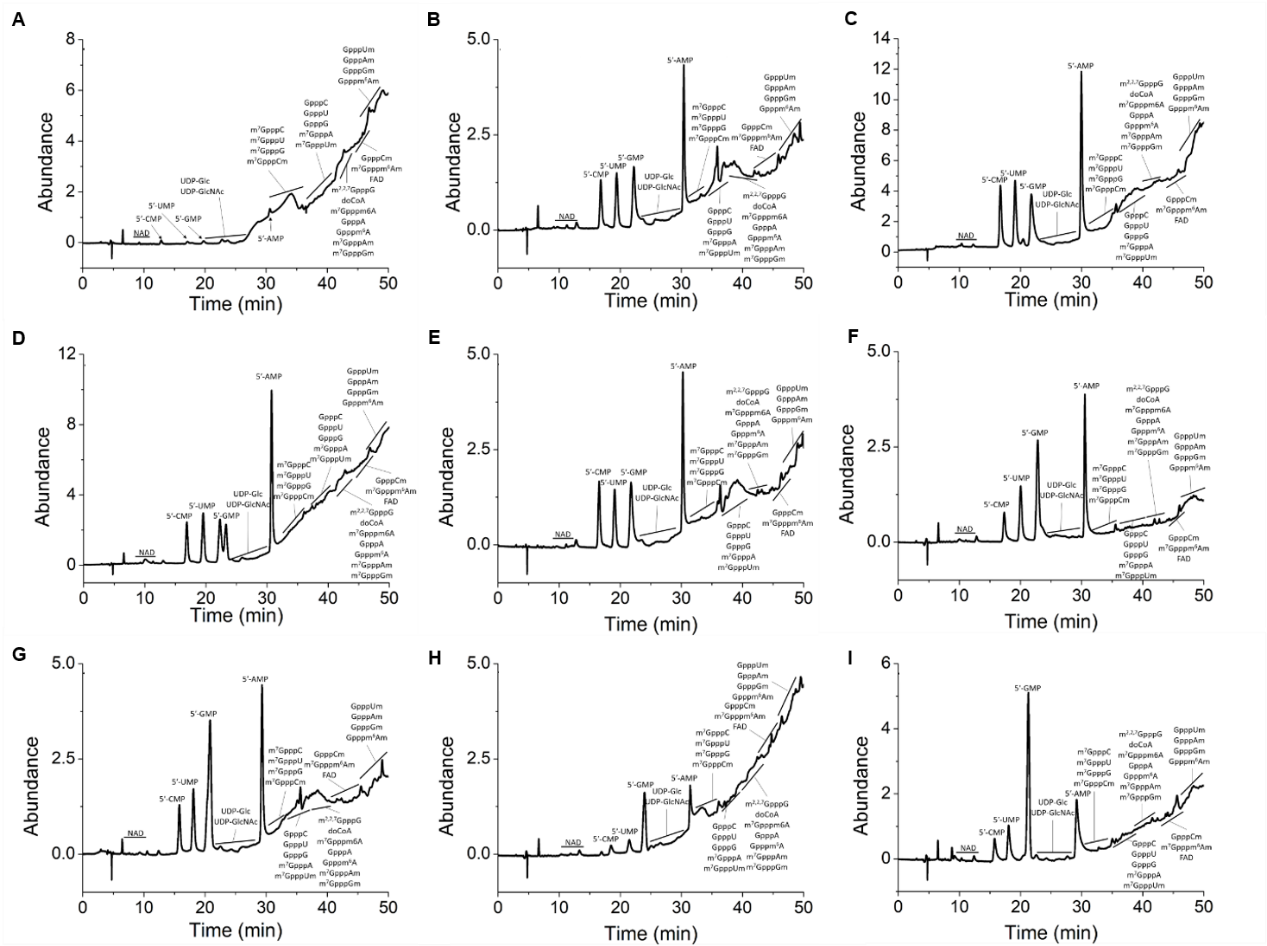


**Figure S3 HPLC trace for the separation of the enzymatic digestion mixture of mRNA.** A: U-2 OS cell; B: cap-modifying enzyme RNMT Knockdown cell; C: modifying enzyme PCIF1 Knockdown cell; D: NaAsO_2_ Stress Granules (As SG); E: Heat shock Stress Granules (HS SG); F: UVA Stress Granules (UVA SG); G: NaAsO_2_ Stress Granule cores (As SG cores); H: Heat shock Stress Granule cores (HS SG cores); I: UVA Stress Granule cores (UVA SG cores)


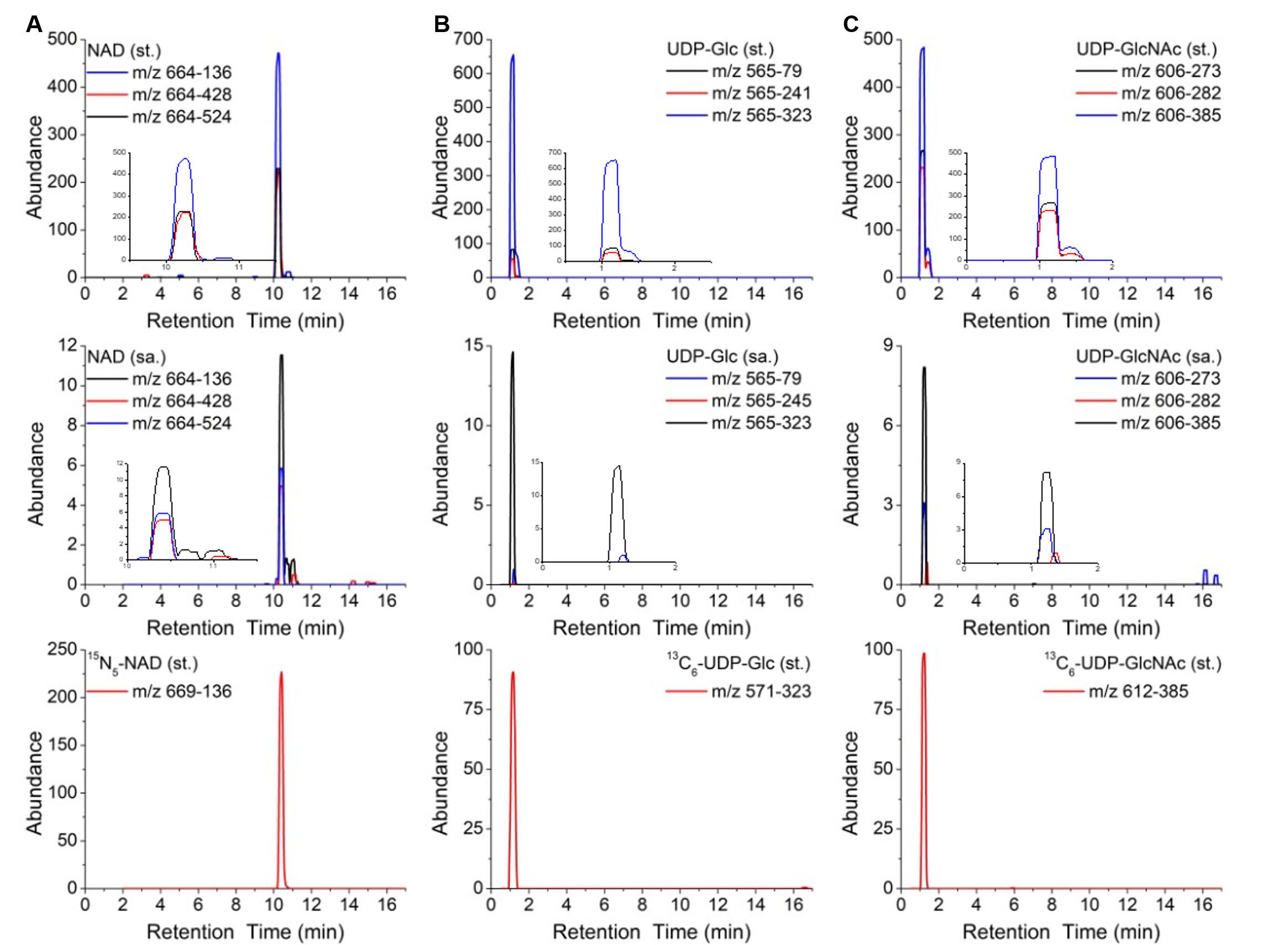

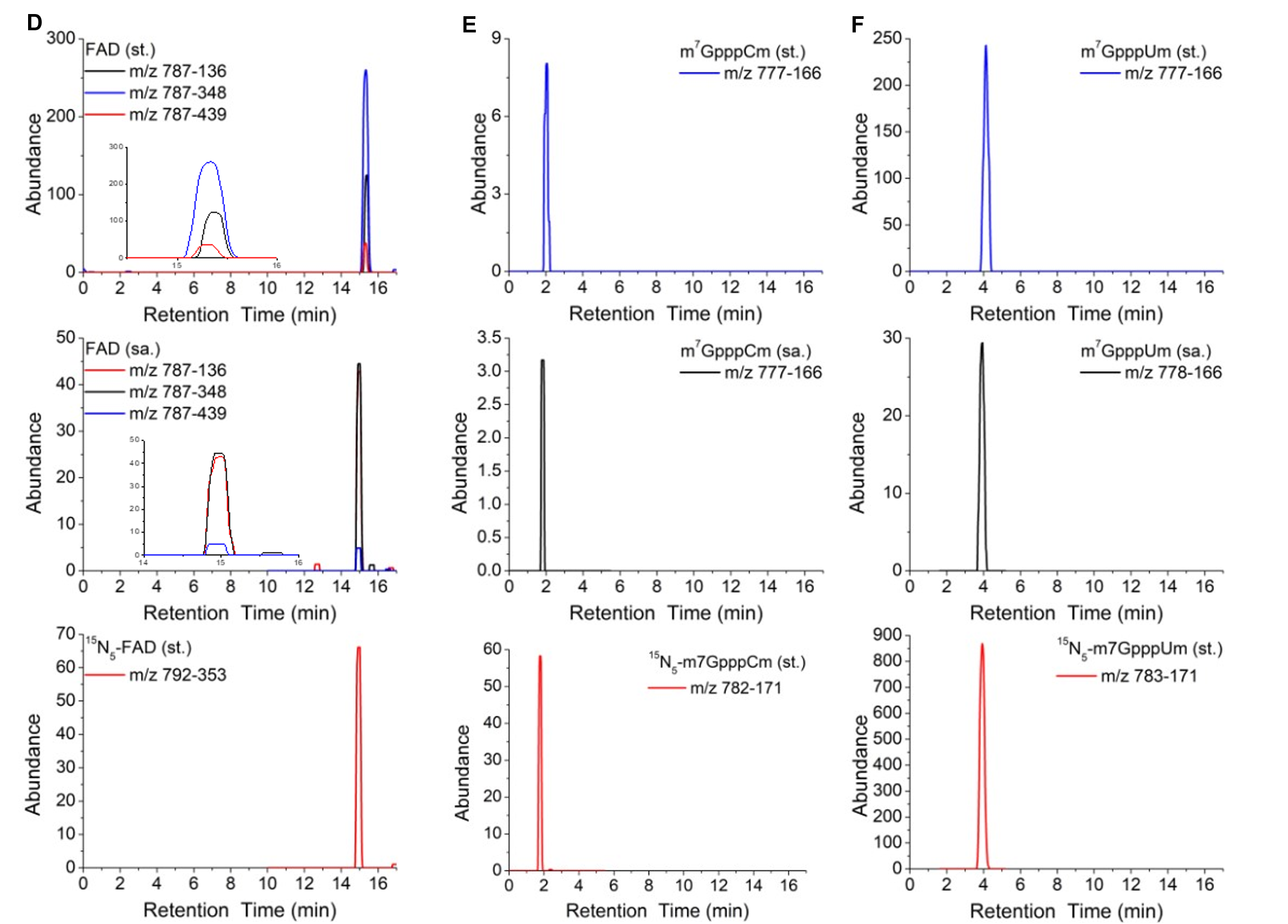

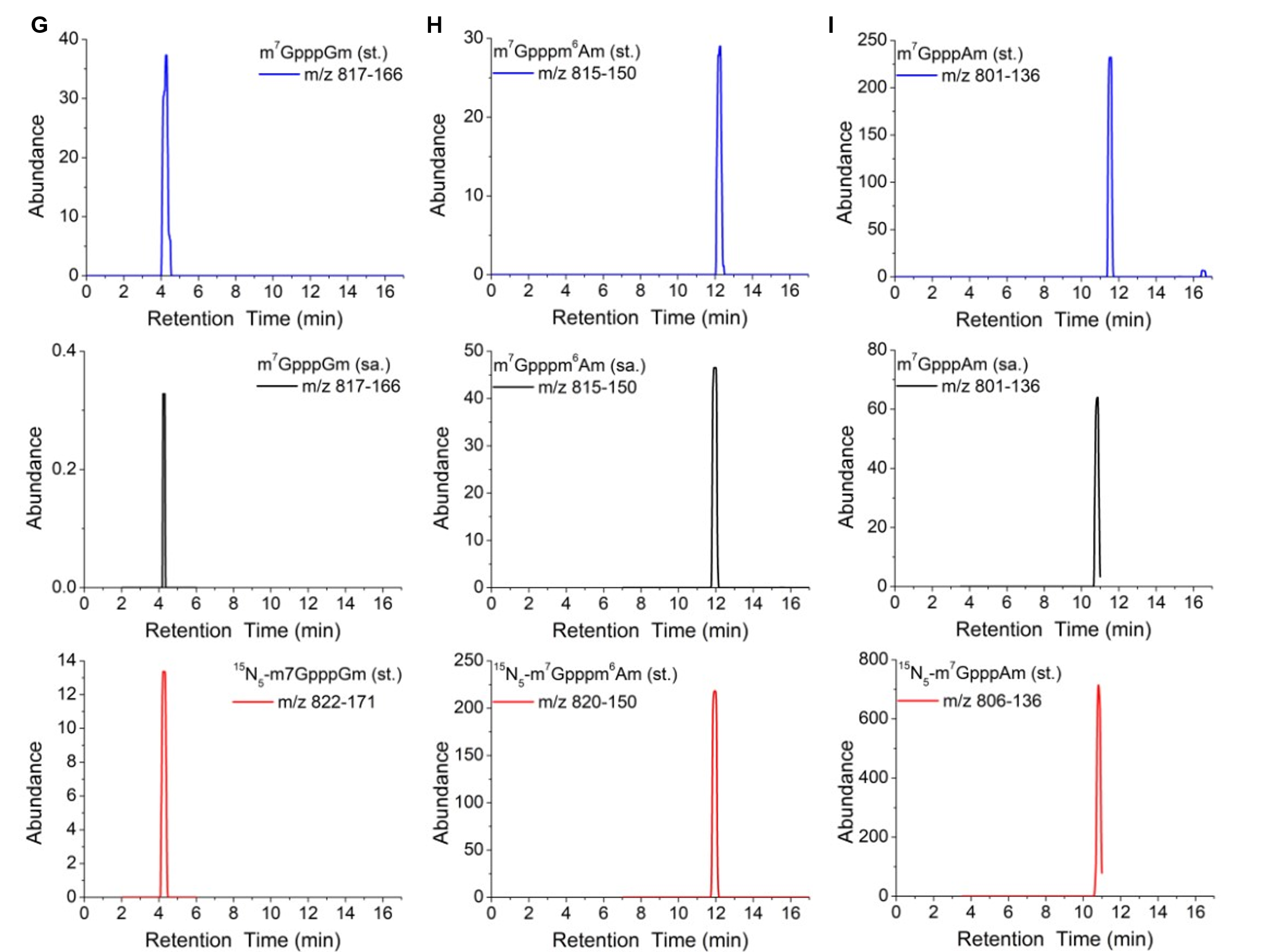


**Figure S4** **Analysis of 5′ cap structures in mRNA by Cap-Quant.** A-I: NAD, UDP-Glc, UDP-GlcNAc, FAD, m^7^GpppCm, m^7^GpppUm, m^7^GpppGm, m^7^Gpppm^6^Am, m^7^GpppAm; showing HPLC elution profiles and MS/MS transitions (*m/z* X→Y) for unlabeled pure standard (top), the mRNA sample (middle), and isotope-labeled standard spiked into the mRNA sample (bottom)


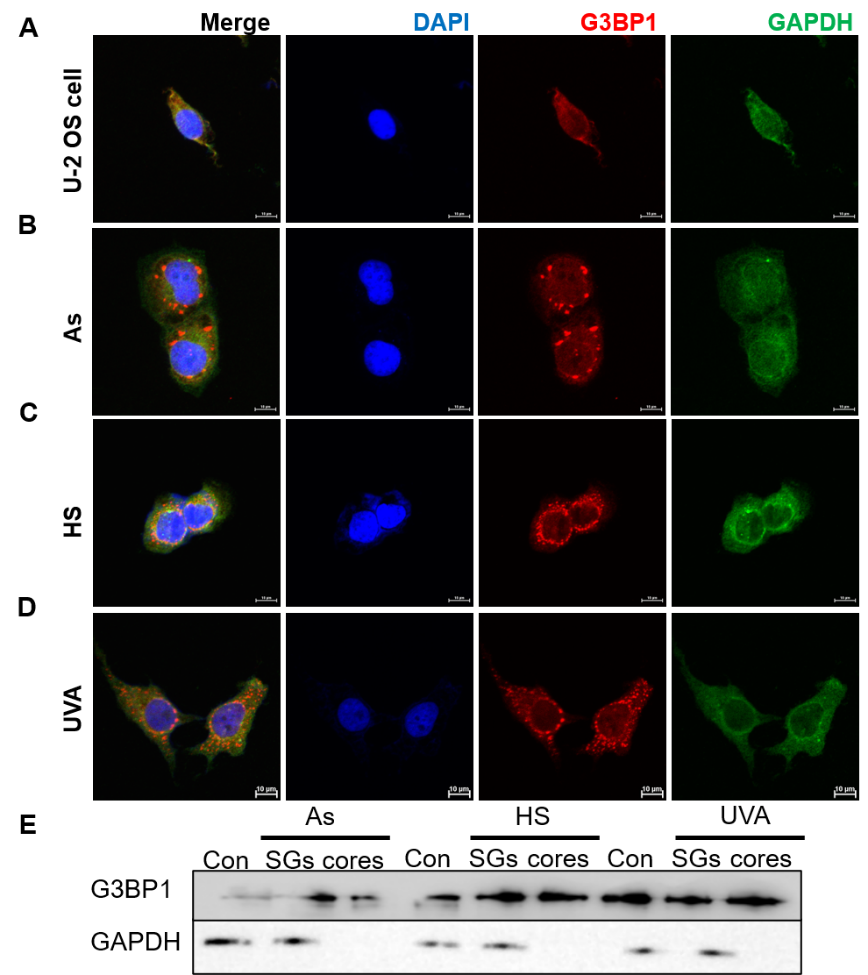


**Figure S5** **Confocal immunofluorescence analysis of untreated or post-stress-treated U-2 OS cell (housekeeping protein glyceraldehyde-3-phosphate dehydrogenase GAPDH labeled with Green) was performed using the G3BP1 protein (red) and SG cores purification assay.** A: U-2 OS cells; B: U-2 OS cells after As (0.5 mM NaAsO_2_) treatment for 1 h; C: U-2 OS cells after heat shock stress (45 ℃) treatment for 1 h; D: U-2 OS cells after 2 h of ultraviolet radiation (UVA) treatment. (The scale bar in the figure represents 10 μm); E: WB assay for SG cores purification.


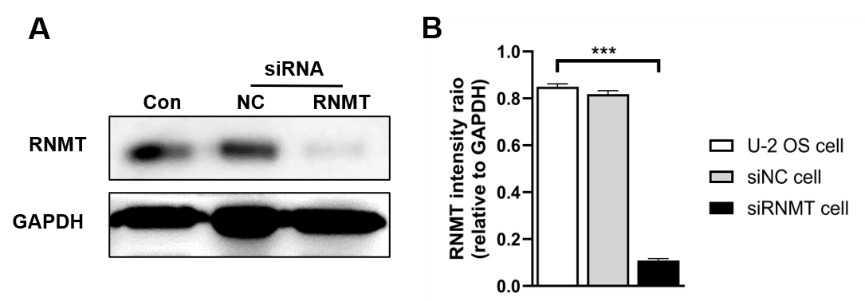


**Figure S6 Detection of RNMT knockdown efficiency in cells by Western Blot.** A: Western blot for protein expression levels of RNMT; B: Quantification of protein bands using ImageJ software.


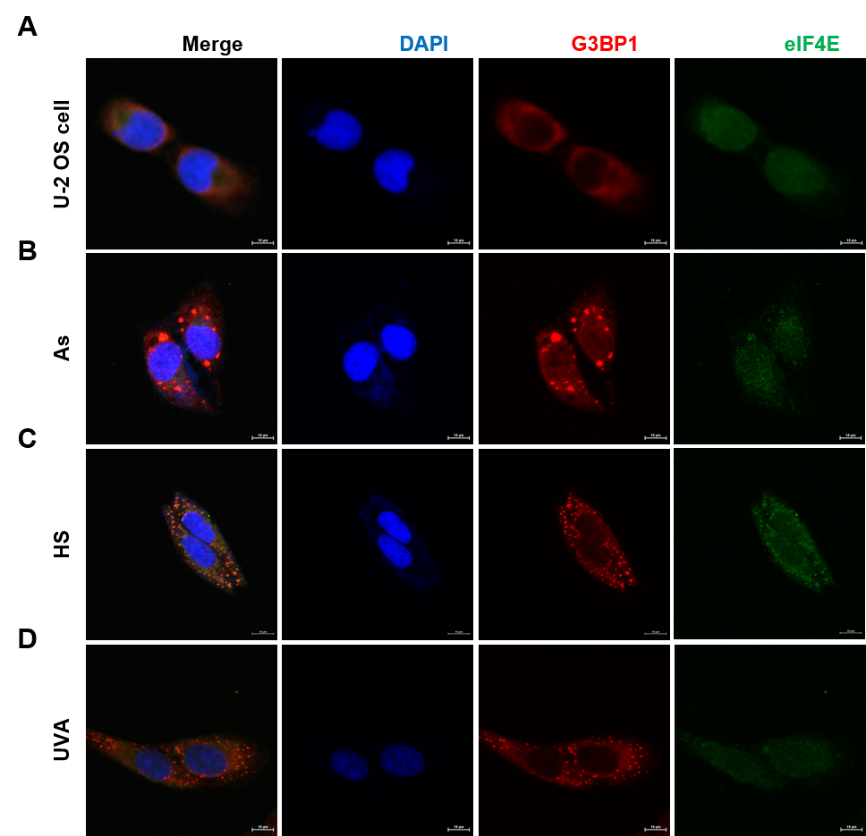


**Figure S7 Confocal immunofluorescence analysis of untreated or post-stress-treated U-2 OS cells using G3BP1 protein (red) (cap-binding protein eIF4E labeled with Green).** A: U-2 OS cells; B: U-2 OS cells after As (0.5 mM NaAsO_2_) treatment for 1 h; C: U-2 OS cells after heat shock stress (45 ℃) treatment for 1 h; D: U-2 OS cells after 2 h of ultraviolet radiation (UVA) treatment. (The scale bar in the figure represents 10 μm)

~~
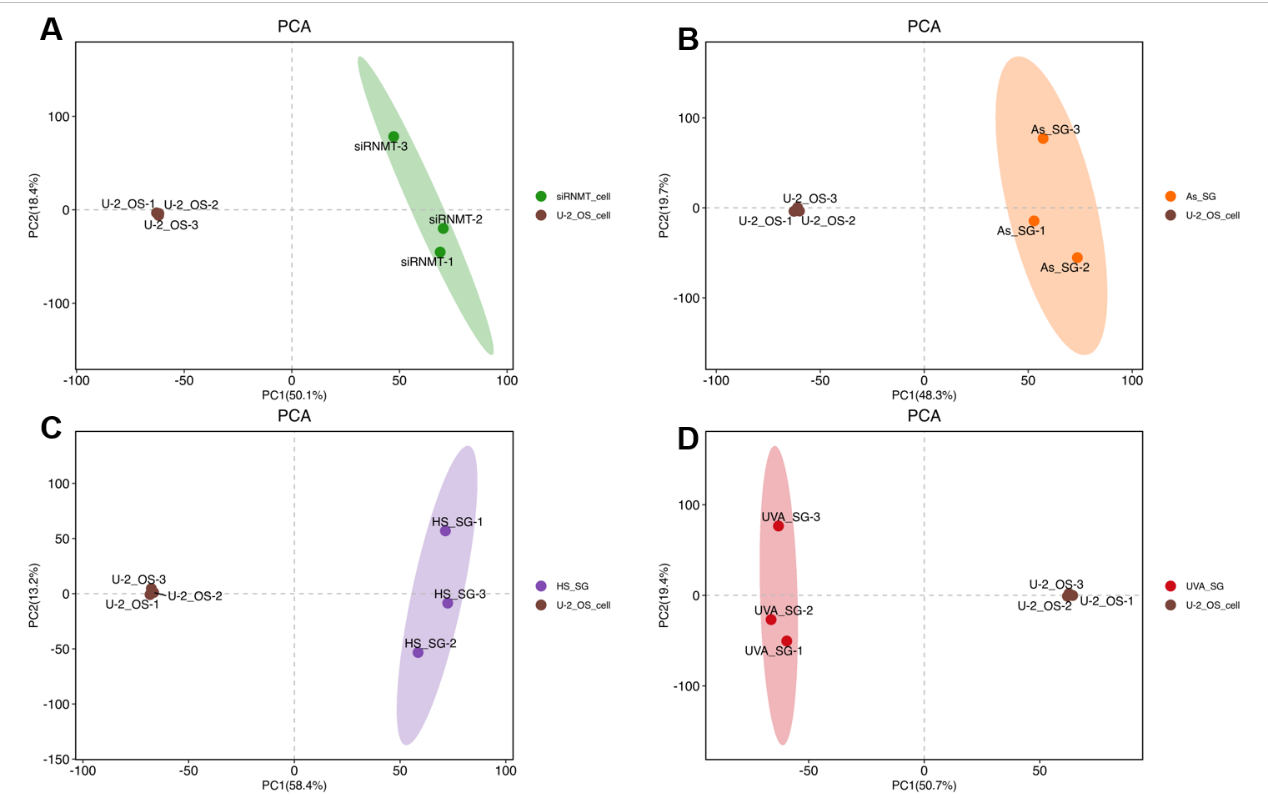
~~

**Figure S8 PCA analysis of repeatability and between-group differences within different experimental groups.** A: RNMT knock down cell -vs- U-2 OS cell; B: As SG -vs- U-2 OS cell; C: HS SG -vs- U-2 OS cell; D: UVA SG -vs- U-2 OS cell.


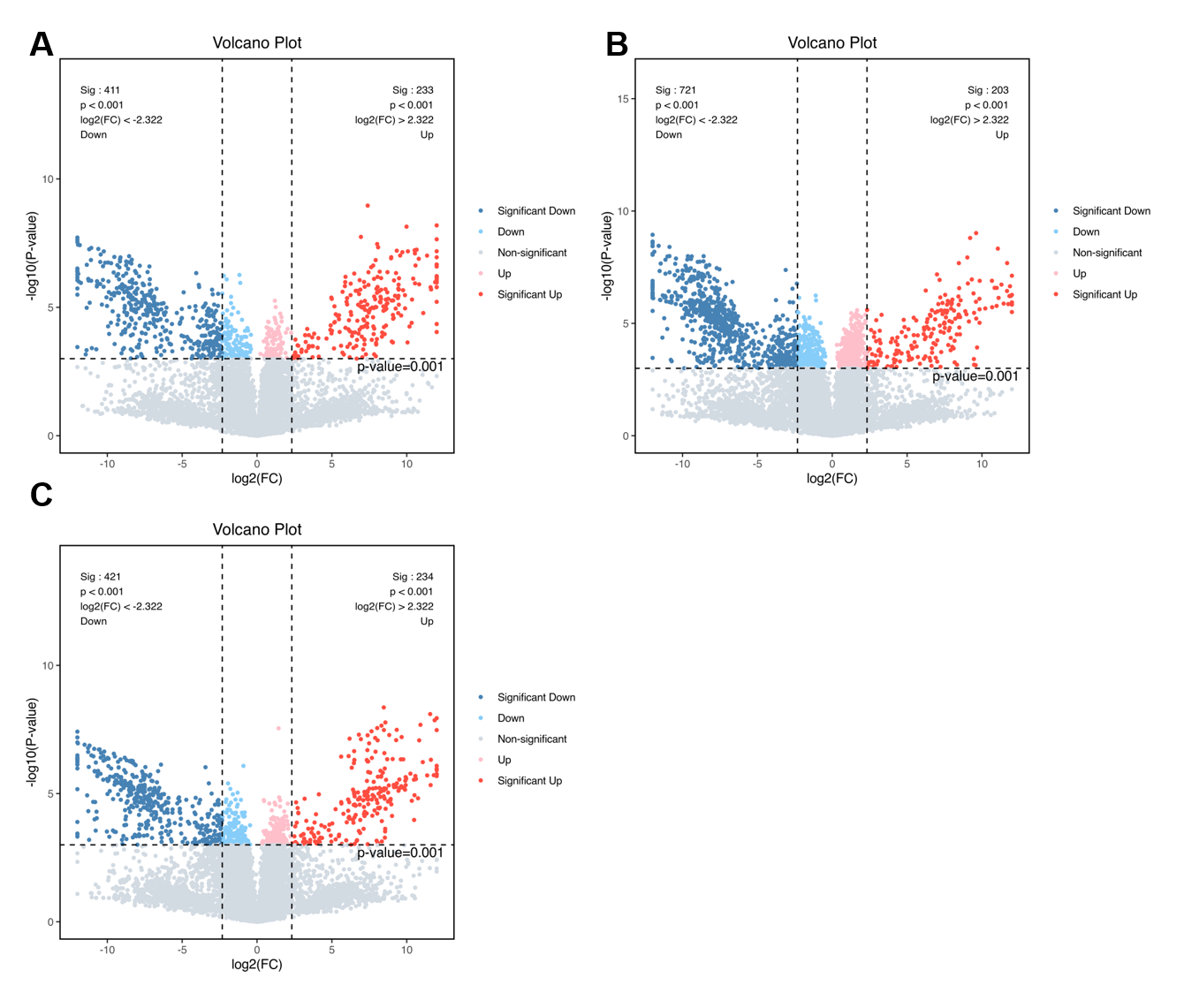


**Figure S9 Proteomics test PCA analysis DEP.** A: As SG -vs- U-2 OS cell; B: HS SG -vs- U-2 OS cell; C: UVA SG -vs- U-2 OS cell.


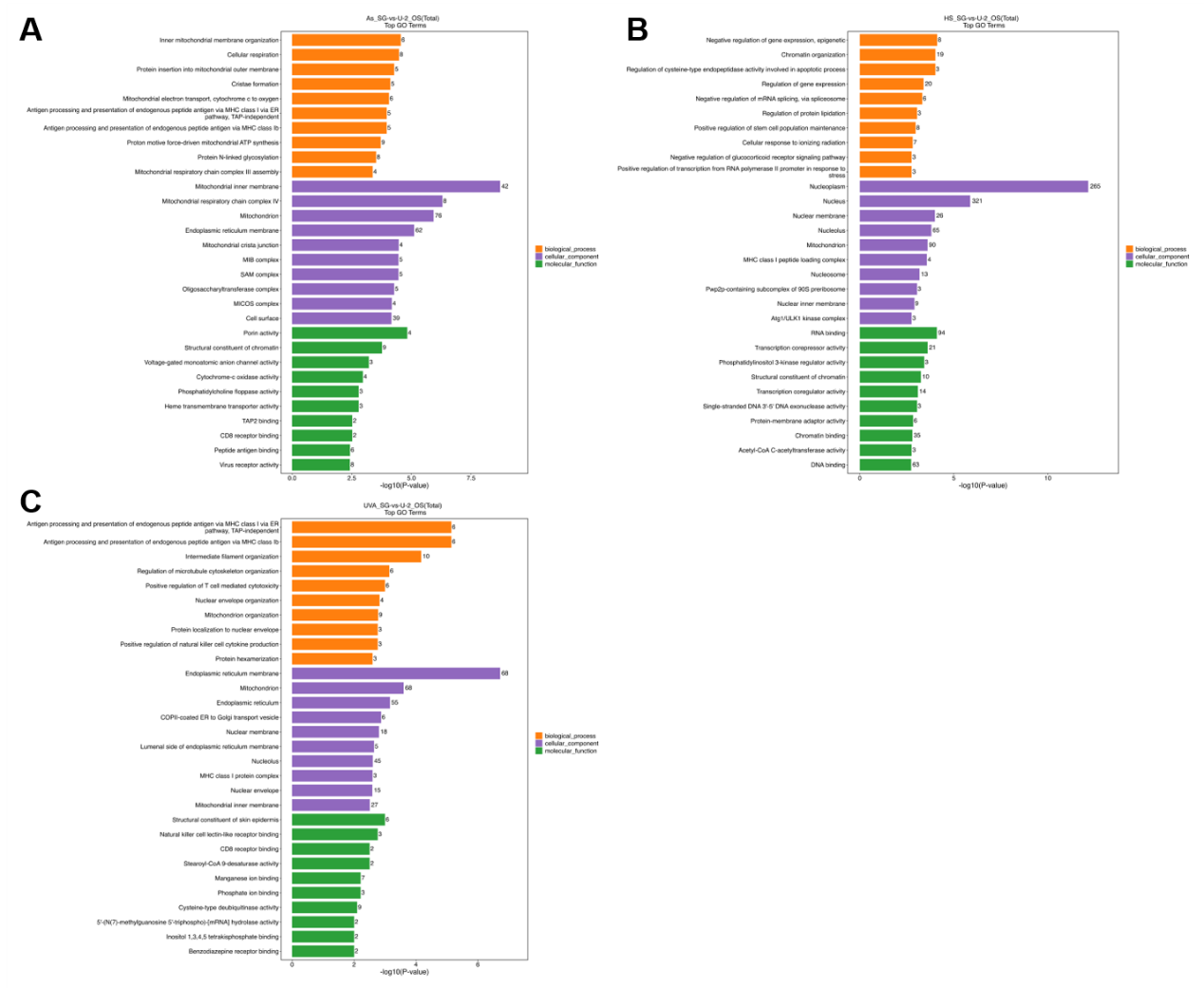


**Figure S10 Top 30 pathways for DEP GO enrichment in proteomics.** A: As SG -vs- U-2 OS cell; B: HS SG -vs- U-2 OS cell; C: UVA SG -vs- U-2 OS cell.


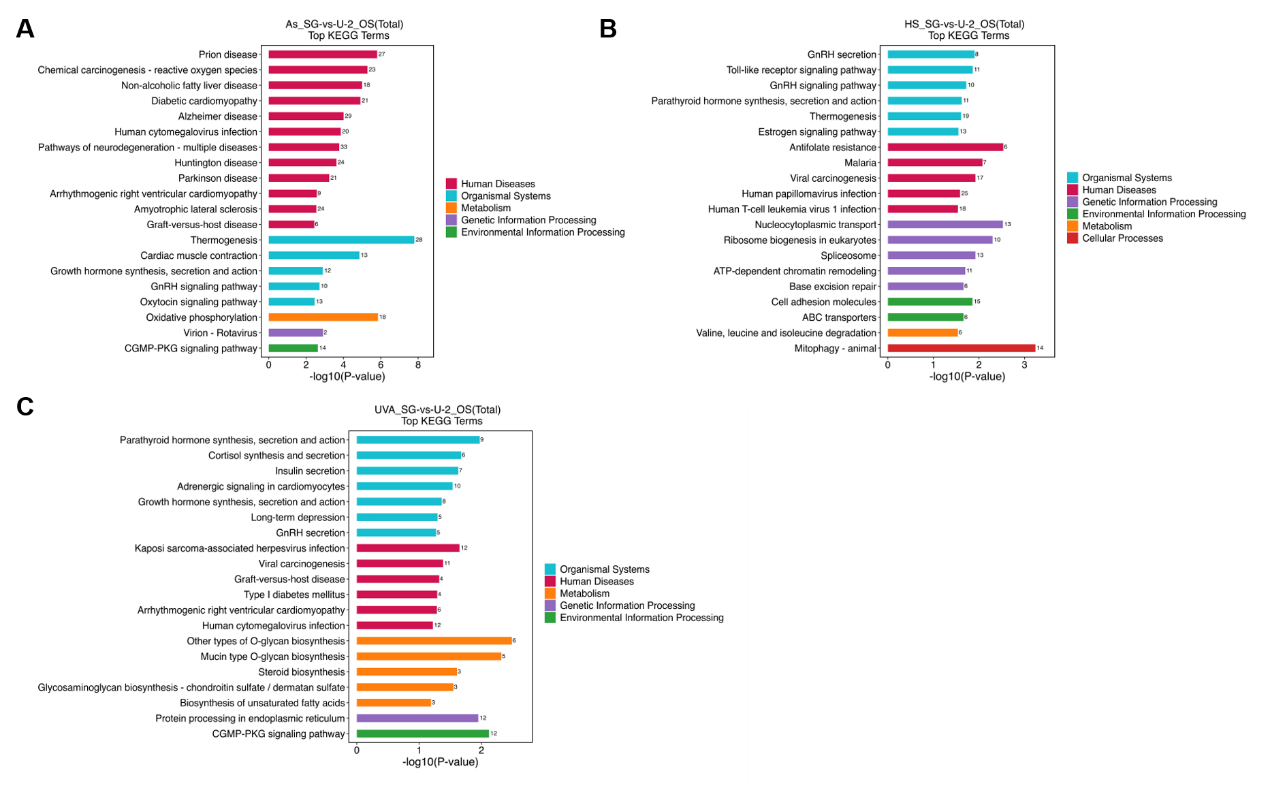


**Figure S11 DEP ratio analysis of KEGG pathway enrichment of the first 20.** A: As SG -vs- U-2 OS cell; B: HS SG -vs- U-2 OS cell; C: UVA SG -vs- U-2 OS cell.


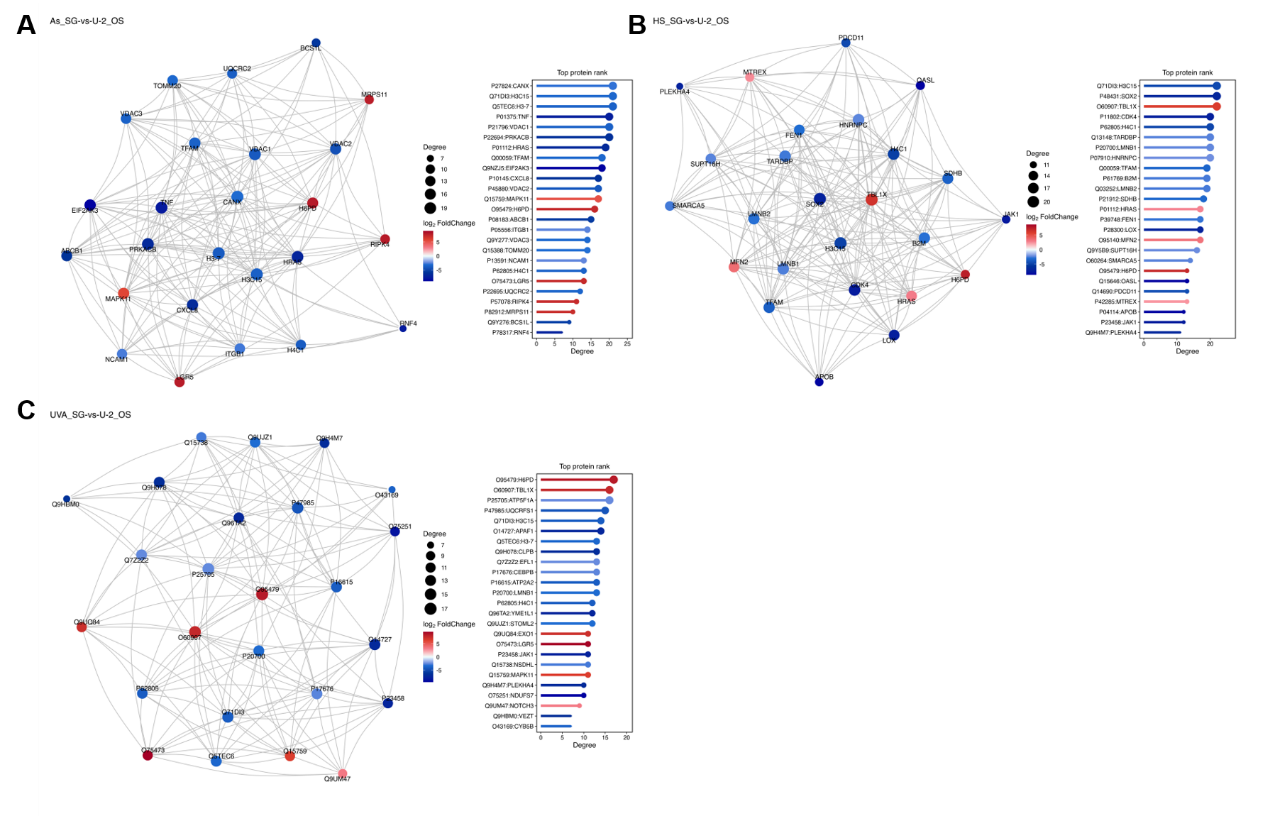


**Figure S12 Proteomics DEP differential protein interactions (Top25 connectivity).** A: As SG -vs- U-2 OS cell; B: HS SG -vs- U-2 OS cell; C: UVA SG -vs- U-2 OS cell.

~~
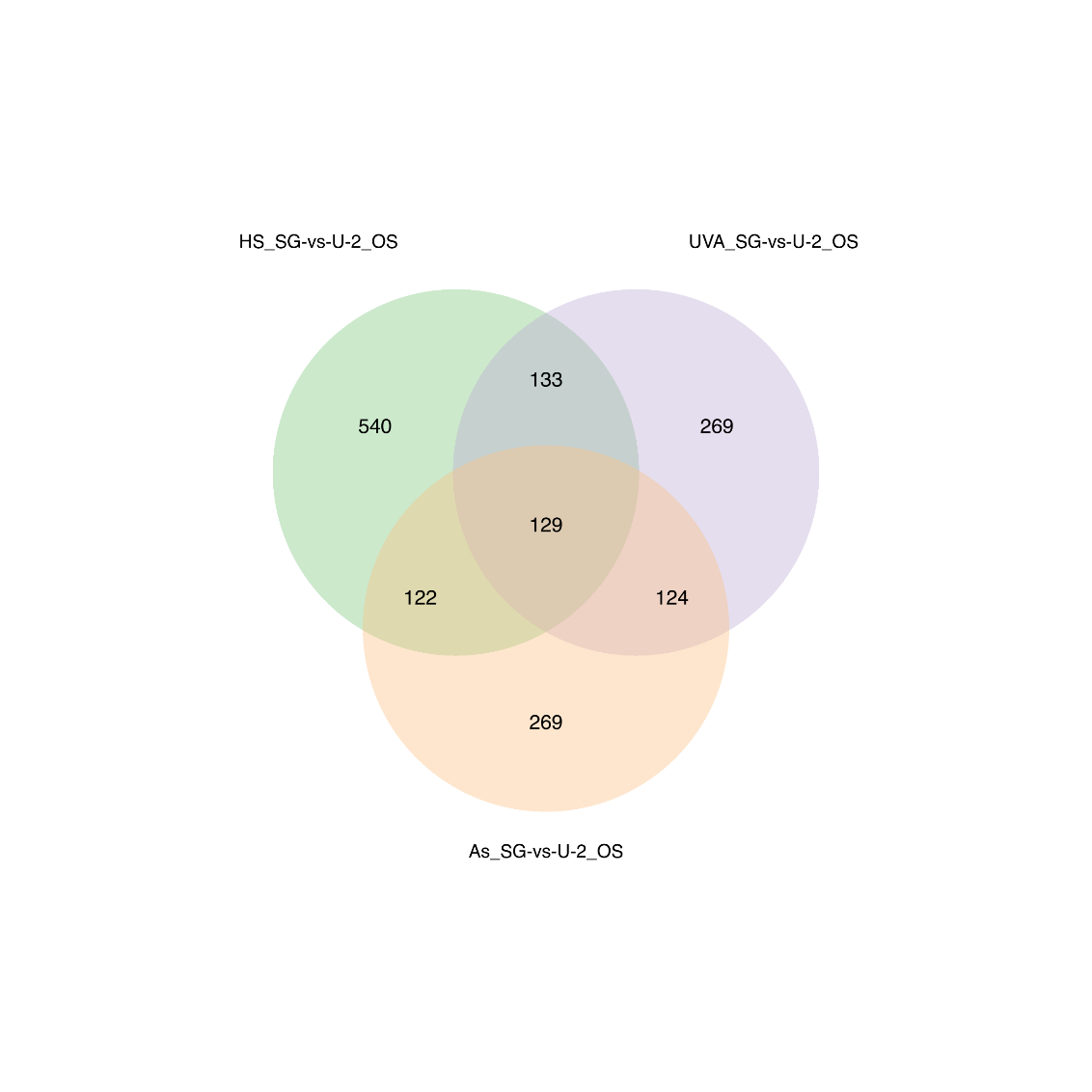
~~

**Figure S13 Venn analysis of DEPs reveals shared and condition-specific signatures across SG.**
